# Bioinspired spatiotemporal control of microhelix formation and actuation

**DOI:** 10.1126/sciadv.aed9514

**Published:** 2026-06-05

**Authors:** Xin Hu, Benjamin R. Greenvall, Eric Chia, Zhaojie Zhang, Stephen S. Nonnenmann, Alfred J. Crosby

**Affiliations:** ^1^ Department of Polymer Science and Engineering, University of Massachusetts Amherst, Amherst, MA 01003, USA.; ^2^ Department of Mechanical and Industrial Engineering, University of Massachusetts Amherst, Amherst, MA 01003, USA.; ^3^ Department of Chemistry, University of Massachusetts Amherst, Amherst, MA 01003, USA.

## Abstract

Helical structures provide critical functions in structural stability, locomotion, and mechanical flexibility. Among the helical structures, the dynamic coiled tendril formation in climbing plants upon contact with support structures inspires the development of numerous helix-based actuators and soft robotics. However, achieving precise spatiotemporal control over helix formation and actuation at the microscale remains a challenge. We introduce a materials system in which the spatial location and dynamics of helix formation are governed by the intrinsic bending resulting from the differential swelling of polyacrylic acid copolymer hydrogels, with electric fields serving as the primary control for electroosmotic flow–induced swelling/deswelling phase transitions. By manipulating electric field polarity and using patterned substrates, we achieve reversible spatiotemporal control over helix formation and actuation. The swelling/deswelling mechanism enables the applications of rotary actuation and controlled microsphere capture-release. Our approach represents a notable advancement in the precise dynamical control of helix formation, opening avenues for the development of sophisticated microactuators and artificial muscle systems.

## Teaser

Polyelectrolyte hydrogel microhelices undergo reversible spatiotemporal transformations under applied electric fields.

## INTRODUCTION

Helical structures pervade nature at both microscopic and macroscopic scales, serving diverse biological functions (*1*–*4*). At the molecular level, DNA molecules adopt double-helical configurations to enhance structural stability (*5*). In the microbial world, numerous bacteria, such as *Escherichia* and *Salmonella* species, use helical flagella as propulsive engines to navigate their environments (*6*). On a larger scale, climbing plants develop helical tendrils that connect their stems to supporting structures, providing resilience against mechanical loads and wind forces. Plant tendrils represent a particularly fascinating case, distinct from the intrinsically twisted helices found in DNA and bacterial flagella. These plant structures coil through intrinsic curvature alone, distinctly allowing both left- and right-handed spirals within a single fiber, connected by a characteristic reversal point known as a perversion (*7*–*9*). While researchers have successfully replicated the complex morphologies of these tendrils in synthetic systems (*10*, *11*), dynamic reversible control over their formation remains an elusive challenge.

**Fig. 1.**
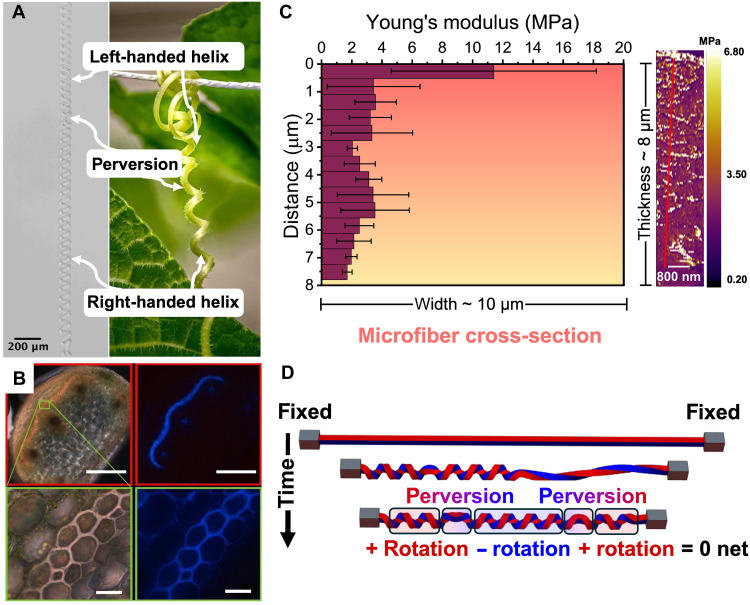
Coiling mechanism of PAA microfibers. (**A**) The PAA microfibers (left) coiled into helices with both left and right handedness connected by the perversions, similar to cucumber tendrils (right). (**B**) Microscopy analysis of a cucumber tendril cross section via darkfield (top left) and ultraviolet (UV) fluorescence (top right) imaging. A distinct region of high lignification is evident, characterized by intense blue fluorescence under UV excitation. Scale bars, 100 μm (primary images, red frames). Corresponding high-magnification views of the highlighted areas (green boxes) are provided below. Scale bars, 10 μm. [Reproduced from Gerbode *et al.*, *Science*, DOI:10.1126/science.1223304 (2012), AAAS (*22*).] (**C**) Atomic force microscope (AFM) indentation measurements from the deswelled microfiber’s surface treated in RIE revealed a gradient of decreasing elastic moduli. (**D**) Simulation of microfiber coiling with both ends fixed and no initial twist demonstrated that left-handed and right-handed helical configurations coexist and topologically cancel out, resulting in a net-zero chirality.

Tendrils in climbing plants exemplify a remarkable two-phase sequence of attachment and upward growth. Initially, tendrils undergo circumnutation-exploratory movements that seek supportive structures (*12*, *13*). Thigmotropism, response to contact, triggers rapid coiling, securing the tendril to the support (*12*, *13*), followed by the winding of the tendrils between the contact and the stem. The tendril’s anchoring process has inspired innovations in soft robotics, including grippers and climbing robots (*14*–*16*), while researchers have replicated the winding mechanism to design shape-morphing actuators capable of reversible deformations (e.g., bending, twisting, and coiling) (*10*, *17*–*19*). However, natural tendrils exhibit great precision in the two-phase growth: Their coiling is spatiotemporally controlled, initiating only after contact stimulus and forming both straight and coiled segments (Fig. 1A). Emulating this precise, stimulus-responsive control could advance the performance of artificial actuators and soft robots in complex environments.

Here, we use a classically described mechanism, the onset of a volume phase transition within polyelectrolyte hydrogels induced by an electric field, to control both the dynamic coiling and spatial organization of microhelices, similar to behaviors observed in plant tendrils. The system’s design relies upon engineered differential swelling within the microfibers to mirror the natural strain mechanism in tendrils while taking advantage of electrically controlled swelling/deswelling transitions to spatiotemporally control helical formation. The coiling/uncoiling process was used to rotate attached microspheres and enable controlled microsphere release and capture, demonstrating the system’s potential as a rotary actuator and for targeted delivery applications. This approach achieves spatiotemporal helical control that surpasses previous engineered systems at the microscale and opens pathways for advanced technologies, such as microactuators, microengines, and artificial muscles.

## RESULTS

### Microfiber coiling mechanism

In plant tendrils, interior layers undergo a transformation after contact with support, developing a stiff, lignified gelatinous fiber (g-fiber) reminiscent of woody tissue (Fig. 1B). This g-fiber exhibits asymmetric contraction, with enhanced shrinkage on the ventral side compared to the dorsal side, driving tendril coiling into left- and right-handed helices with an intrinsic curvature (*20*–*22*). Harnessing this mechanism of differential strain, we engineered the differential swelling of poly(acrylic acid) (PAA)–based copolymer hydrogel microfibers in water to obtain coils through intrinsic bending. To increase the swelling ratio of PAA hydrogels in water (*23*), acrylic acid (AA) is typically copolymerized with alcohols, including 2-hydroxyethyl methacrylate (*24*, *25*) and 4-hydroxybutyl acrylate (4-HBA) (*26*, *27*); here, 4-HBA was selected. The presence of ionic AA groups provides electric field–responsive swelling/deswelling behavior, enabling controlled coiling and uncoiling. After fabrication through nanoimprint lithography (NIL) (*28*), the microfibers were simultaneously separated and modified with reactive ion etching (RIE) (fig. S2). As reactive species (e.g., radicals and ions) with high energy diffused into the microfiber through its surfaces, polymer chains underwent scission and associated cross-linking reactions, thus creating regions with increased modulus. With the limited diffusion of the reactive species, a gradient of the elastic modulus was formed perpendicular to the thickness direction (*29*, *30*). The application of RIE to create gradient moduli in cross-linked networks was established in our previous work (*28*), which systematically examined the influence of RIE parameters and provided quantitative predictions of gradient microfiber bending behavior. This gradient change in mechanical properties was verified through atomic force microscope (AFM) indentation tests at different depths (Fig. 1C). As the g-fiber’s asymmetric contraction drives natural coiling, the asymmetric swelling occurred because the stiffened upper region demonstrated restricted swelling relative to the bottom nonetched part. Upon reaching a critical swelling ratio, the top surface began to fracture because of constrained expansion. Continued swelling after cracking generated greater asymmetric strain, thereby inducing microfiber coiling (fig. S3). During coiling, the osmotic pressure difference from asymmetric swelling caused the bending of the microfiber. To further examine this mechanism, we prepared a short microfiber by razor blade cutting and allowed it to swell in water with both ends free. An average coiling diameter of ~90 ± 5.60 μm was measured across three segments from three different microfibers, consistent with the coiling diameter observed for long microfibers with both ends fixed (~79 ± 6.28 μm). This agreement confirms that microfiber coiling in this system is governed by beam bending (fig. S4). Assuming that the osmotic pressure difference controls the scaling of the tensile stress that develops within the microfiber, the coiling curvature will scale as (*31*)

$$\kappa \propto \frac{∆\pi \;}{E_{\text{avg}}}$$ (1)

where ∆π is the osmotic pressure difference induced by differential swelling, $E_{\text{avg}}$ is the averaged elastic modulus (mean modulus across the thickness, D; $E_{\text{avg}}=\frac{\int_{0}^{D}E\;\left(x\right)\;\mathrm{dx}}{D}$), and κ is the coiling curvature.

Notably, before being immersed in water, the two ends of the microfibers were fixed to an indium tin oxide (ITO) substrate with epoxy adhesive. This configuration kept the microfiber close to the electrode for a fast response (*32*, *33*). Without this constraint, a microfiber with one or both ends free would extend in the *z* direction due to gravity and form helical coils in three-dimensional (3D) space rather than the planar coils formed in the *x*-*y* plane with both ends fixed (fig. S5). In addition, fixing both ends restricted twisting at the terminals. Consequently, during microfiber coiling, both right-handed and left-handed helices were generated alternately to maintain zero net twist, connected by perversions—a morphology that mimics plant tendrils. However, perversions can also arise from the large aspect ratio of the fiber cross section, independent of the two-end fixed boundary conditions (*34*).

To understand the perversion formation mechanism, we conducted elastic rod simulations under three different conditions: an initially straight rod with two ends fixed (analogous to experiments), an initially straight rod with one end fixed, and an initially pretwisted rod with two ends fixed. Comparing the straight rod simulations, the perversions emerged only in the initially straight rod with two-end fixed case, demonstrating that a two-end fixed boundary condition was necessary for perversion formation. Notably, when pretwisted rods with fixed ends were tested, no perversions appeared, indicating that perversion formation required pure bending as the coiling mechanism instead of twisting (Fig. 1D and Supplementary Text). This biomimetic approach effectively translated nature’s differential strain mechanism into artificial systems through carefully engineered asymmetric swelling properties of hydrogel microfibers.

### Electric field responsiveness working principle

**Fig. 2.**
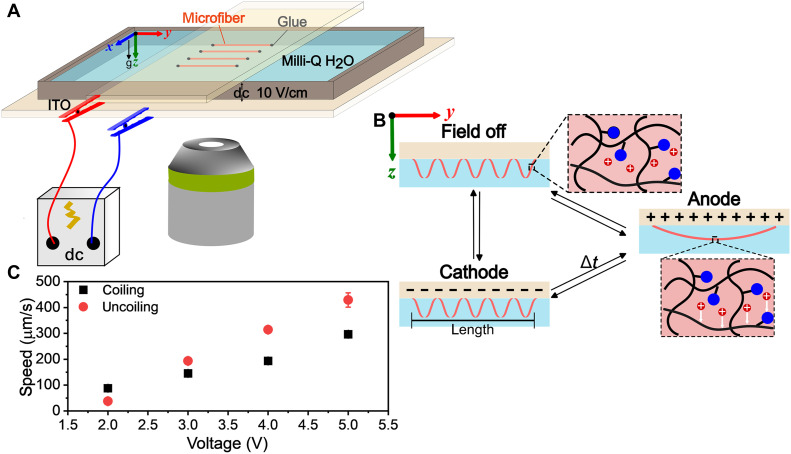
Electric coiling mechanism of PAA microfibers. (**A**) Experimental setup from top to bottom: ITO glass with imprinted microfibers, spacer, bare ITO glass, and microscope for morphology observation. (**B**) Schematic of the coiling and uncoiling transition in electric fields. In the zoomed-in polymer structure, blue spheres represent ─COO^−^, red spheres represent H^+^, and black lines represent the ─C─C─ backbone from AA and 4-HBA monomers. For simplicity, pendant hydroxyl butyl groups are not shown. When the microfiber contacted the anode, proton migration toward the cathode generated electroosmotic flow, causing the microfiber to deswell and straighten. Conversely, at the cathode, water flowed back into the polymer matrix, causing the microfiber to swell and coil. (**C**) The coiling and uncoiling speed of the microfiber, characterized by the length of the microfiber over the time for the transformation, increased linearly with the input voltage due to faster proton drift under higher electric fields.

The electro-responsive behavior of the PAA-based microfibers was investigated in a Milli-Q water bath. The setup consisted of the ITO glass bearing the microfibers as one electrode (top) and a bare ITO glass as the counter electrode (bottom). The two glass slides were separated by a 2-mm-thick 3D-printed frame, connected to a 2-V dc power source (Fig. 2A). The spatiotemporal control over helix formation is achieved through electrically driven swelling/deswelling phase transitions (*35*–*37*). Specifically, at the cathode, the microfibers swelled and coiled; the resulting coiling states were governed by the hydrogel composition within the microfibers and could differ from those observed in the absence of an electric field, as discussed in detail later. Conversely, at the anode, they deswelled and straightened.

Several mechanisms have been proposed to explain electrochemically controlled swelling and deswelling in polyelectrolyte hydrogels, including the electroosmotic mechanism (*33*), the Coulomb mechanism (*38*, *39*), the electrochemical mechanism (*40*), and the dynamic enrichment/depletion mechanism (*41*, *42*). As these systems involve complex interactions among electrophoresis, electrochemistry, and electrostatics, the dominant mechanism depends on experimental conditions, and multiple mechanisms may act simultaneously. In our system, Milli-Q water was used as the solvent instead of salt solutions; therefore, the dynamic ion enrichment/depletion effects were minimized. In addition, cyclic voltammetry (fig. S6) revealed that electrolysis occurred between −2 and 2 V. Therefore, pH changes could influence the swelling behavior of microfibers. However, the microfiber configuration and dimensions were maintained for up to 4 hours, suggesting that possible time-dependent pH changes associated with electrolysis did not play a dominant role in microfiber swelling/deswelling (fig. S7). We conclude that the electrically driven swelling/deswelling transitions were primarily governed by electroosmotic flow within the hydrogels. Since both ends of the microfibers were fixed to the substrate, the microfibers remained in close proximity to the electrodes. When positioned near the anode, the mobile protons from AA groups within the PAA migrated toward the cathode due to Coulomb forces, while the polyions (carboxylate ions) on the polymer molecules remained fixed. Simultaneously, the water molecules around the protons were dragged toward the cathode and generated electroosmotic flow (*32*, *33*, *43*, *44*), which caused the microfiber to deswell and eliminated osmotic pressure differences across the microfiber thickness. With minimal osmotic pressure difference, the bending curvature approached zero, corresponding to a straight, unbent microfiber (Eq. 1). Conversely, when the microfiber is near the cathode, water molecules flowed back into the PAA hydrogel matrix, and osmotic pressure differences caused the microfiber to reswell and induce helix formation (Fig. 2B). This electric field–responsive system enables reversible switching between two distinct configurations—straight when proximate to the anode and helical when near the cathode Fig. 2B).

**Fig. 3.**
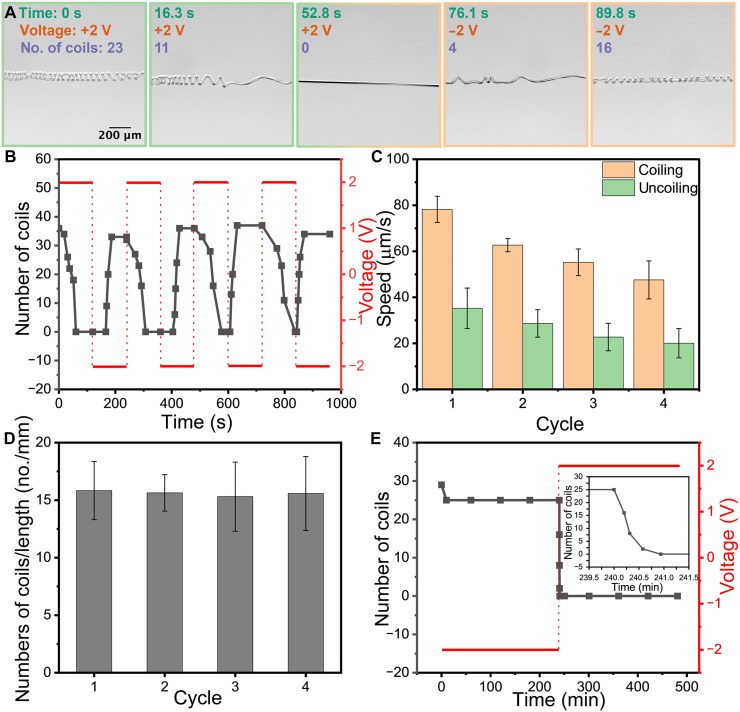
Temporal regulation of helical formation. (**A**) Time-lapse showed helix dissolution at the anode and reformation upon field reversal when the polarity of the field is sequentially changed. All the images had the same scale bar. (**B**) The monitored number of coils while the electric field polarity was switched four times to quantify the transition kinetics and statics. (**C**) The kinetics during the coiling/uncoiling transformation. The speeds during each cycle decreased with an increasing cycle number, which can be attributed to proton depletion within the hydrogel matrix following repeated cycling. (**D**) The number of coils at equilibrium at the cathode remained consistent across cycles, demonstrating the reproducibility of the microfiber configuration. (**E**) The stability of both straight and coiled microfiber configurations over 4 hours demonstrated that these states can be maintained and controlled exclusively through electric field modifications.

**Fig. 4.**
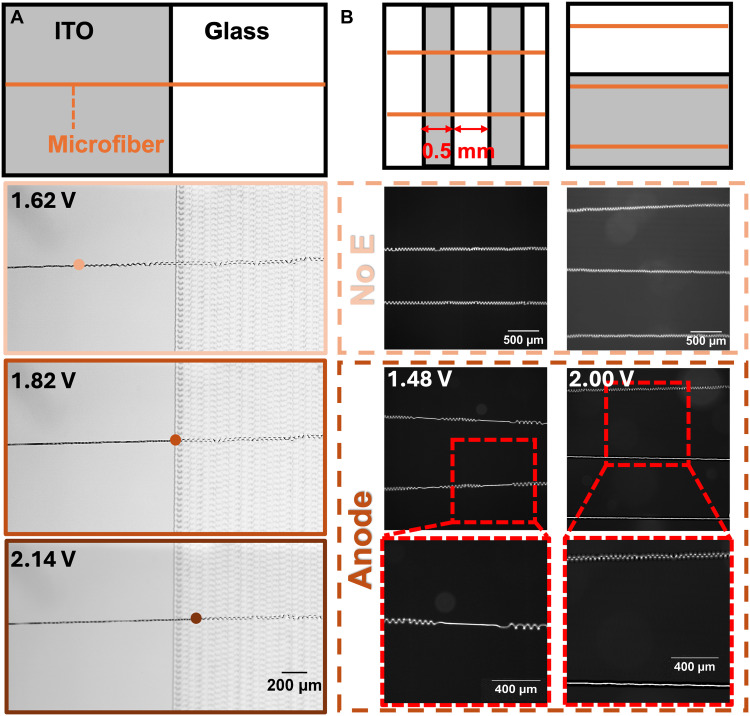
Spatial control of helix distribution. (**A**) The voltage-dependent coiled-to-straight transition. A microfiber spanning an ITO/glass boundary adopted a coiled configuration in water without applied electric fields. When the ITO segment served as the anode, the portion within the electric field began to deswell and transitioned to a straight configuration. The swell-to-deswell transition front shifted toward the glass region with increasing input voltage (the transition points were highlighted with dots). All the images had the same scale bar. (**B**) Spatial distribution control of microfibers positioned at different positions relative to ITO/glass boundaries. Left column: Perpendicular segments within a single fiber by controlling the coiled-to-straight transition in ITO regions. Right column: Parallel positioning. Only microfibers positioned over ITO regions transitioned to straight configurations, while fibers over glass regions remained coiled regardless of input voltage, achieving the distribution of the configurations across multiple microfibers.

A threshold potential is defined when the swelling/deswelling phase transitions occur. However, it varies with system resistance and material properties (*37*, *45*) Consequently, the nonpatterned ITO substrates across all experiments, for example in Fig. 3A, exhibit different thresholds than the patterned ITO substrates featured in Fig. 4. In our system, the threshold voltage for the nonpatterned ITO substrate was found to be around 1.6 V by incrementally increasing the applied voltage, a value independent of electrode spacing. As shown in fig. S8, identical input voltages yielded similar kinetics across different spacings. The applied voltage also governed the kinetics of the coiling and uncoiling transformation, regardless of electrode spacing. However, as the input voltage was increased, the speed increased (as seen in the 2-mm spacing example in Fig. 2C). However, upon reaching a voltage of 2.5 V at 2-mm spacing, electrolysis of water was triggered, generating bubbles that accumulated at the top ITO glass, because of buoyancy, where the microfibers were anchored (fig. S9). These bubbles compromised system performance by altering the electrical resistance and physically impeding the microfibers’ transformations. Therefore, the applied voltage was set as 2 V for subsequent experiments to maintain consistent and reliable coiling. Moreover, the actuation was explored across different microfiber lengths, and consistent coiling and uncoiling behaviors were observed, demonstrating the negligible influence of length scale on the actuation behavior (fig. S10).

### Temporal regulation of helical formation

Figure 3A presents sequential frames from movie S1, demonstrating the continuous transformation of microfiber conformations under alternating field polarity. When the ITO glass supporting the microfiber was set as the anode, the initial coiled structure under the field-off condition progressively unwound and deswelled, driven by electro-osmotic flows. Within ~1 min, the microfiber reached a straightened state, indicating the completion of the deswelling phase transition. Immediately after the transformation, the polarity of the applied electric field was reversed by switching the ITO substrate to the cathode. This reversal triggered an influx of water molecules into the hydrogel network, and the microfiber progressively recoiled into helical structures. During coiling, bending initiated at a random segment location along the microfiber, accompanied by rotation of the entire microfiber. After the first coil formed, this bending propagated along the fiber until the entire microfiber was fully coiled (fig. S11). After coiling, the microfiber continued to swell and reached equilibrium after 5 min (fig. S12).

The reversibility of this transformation was demonstrated for 4 cycles, as shown in Fig. 3B. The microfiber with a 2-mm end-to-end distance was studied by maintaining the polarity of the field for 120-s intervals, while the number of coils was counted during transformation. The transformation speed, defined as the number of coils per unit time, was determined to characterize the kinetics. Analysis revealed a progressive decline in both coiling and uncoiling speeds with each successive iteration (Fig. 3C). This degradation likely resulted from progressive depletion of mobile protons through repeated cycling. As demonstrated previously, mobile protons can electrophoretically migrate away from the gel under applied fields, leaving behind slightly negatively charged gels (*33*, *46*, *47*). This loss of protons may accumulate through sequential cycles. According to the Donnan equilibrium theory (*48*), the resulting decrease in ion concentration difference between gel and surrounding solvents reduced the osmotic pressure—the driving force for swelling/deswelling transitions—which corresponded to the observed decrease in transformation speed. While transformation kinetics showed cycle-to-cycle variation, the ultimate microfiber configurations were highly reproducible. Each transformation cycle resulted in complete straightening with zero coils at the anode and consistent coiling quantified via the number of coils at the cathode (Fig. 3D). As a result, the small amount of depletion of the protons in the gel possibly affected the transitions dynamically more than the swelling/deswelling equilibrated configurations. Significantly, the handedness distribution in the helices and the number of perversions can vary between cycles within a single microfiber. This variability likely arises from cycle-dependent changes in transformation kinetics, offering a potential mechanism for topological control in adaptive applications. Kinetic-dependent topological variance has been reported in other simulations and systems (*49*–*51*), whereas topological invariance has been observed in microhelices of liquid crystalline polymers (*10*, *18*, *52*), core-shell multimaterial fiber (*17*), and bilayer materials (*34*).

The stability of the microfiber configuration was investigated under constant electric fields for 4 hours at hourly intervals after the transformation (Fig. 3E and fig. S7). For the coiled state at the cathode, the number of coils decreased within the first 10 min due to continued hydrogel swelling posttransformation, which increased the microfiber’s dimensions and reduced the number of coils within the 1-mm observation window. Over the subsequent 4 hours while maintaining the field, the number of coils remained constant. Upon setting the ITO substrate as the anode, the microfiber reverted to its straight configuration, with detailed kinetics illustrated in the inset of Fig. 3E. The straight conformation then persisted stably for another 4 hours when in the vicinity of the anode. These results demonstrated that both straight and coiled configurations of the microfibers remained stable over extended periods for up to 4 hours without external stimulation. The reversible transformations were exclusively triggered by polarity switching, enabling on-demand control over helical formation activation and deactivation.

### The spatial distribution control of helices with patterned ITO glasses

The spatial distribution of helices can be controlled through the discontinuous nature of the PAA hydrogel’s swelling/deswelling phase transition, which we exploited via selective substrate patterning. With a glass substrate roughly half coated with ITO and half uncoated (nonconductive), we demonstrated the ability to spatially control the swelling/deswelling ratio within a single microfiber through voltage modulation (Fig. 4A). The microfibers were imprinted to span perpendicularly across the bare glass/ITO boundary and initially exhibited uniform coiling when released in water. When the ITO region served as an anode, the segment of the microfiber exposed to the electric field initiated the deswelling response and unwound, with the extent of unwinding dependent on the applied voltage: At a relatively low voltage of 1.62 V, the deswelling region of microfibers remained within the ITO region; when increased to 1.82 V, the transition extended to the ITO/glass boundary; and at 2.14 V, the deswelling front (highlighted with dots) progressed into the glass region, demonstrating the space-tunable nature of this configuration distribution. In the COMSOL simulation, a potential gradient was found along the gravitational sagging microfiber at the ITO/glass boundary. We hypothesized that as the input voltage increased, more segments of the microfiber were in the region above the threshold voltage for the swelling/deswelling phase transition. Therefore, the transition front shifted toward the glass region with increasing voltage (fig. S13). In contrast, the potential distribution within the ITO region was comparatively uniform, which accounts for the absence of similar discrete transitions observed on nonpatterned ITO substrates.

To demonstrate control over the spatial distributions of helices, we positioned the microfibers in two distinct orientations relative to the ITO/glass boundaries: perpendicular and parallel positioning. In perpendicular positioning (the left column in Fig. 4B), microfibers span multiple ITO/glass boundaries. By applying a 1.48-V voltage, straightening occurred within the ITO regions, producing alternating segments of coiled and straight conformations within individual fibers and indicating the helical spatial control at the single-fiber level. In the parallel positioning, the microfibers were aligned parallel to the ITO/glass boundaries (the right column in Fig. 4B). Straightening occurred only in microfibers overlaying ITO regions, regardless of the applied voltage magnitude. This parallel arrangement enabled spatial control across multiple microfibers. By tuning the ITO patterns and microfiber positioning, the spatial distribution of the straight and coiled microfibers can be programmable at both the single-microfiber and multimicrofiber scales.

### Helical diameter control through hydrogel composition variation

**Fig. 5.**
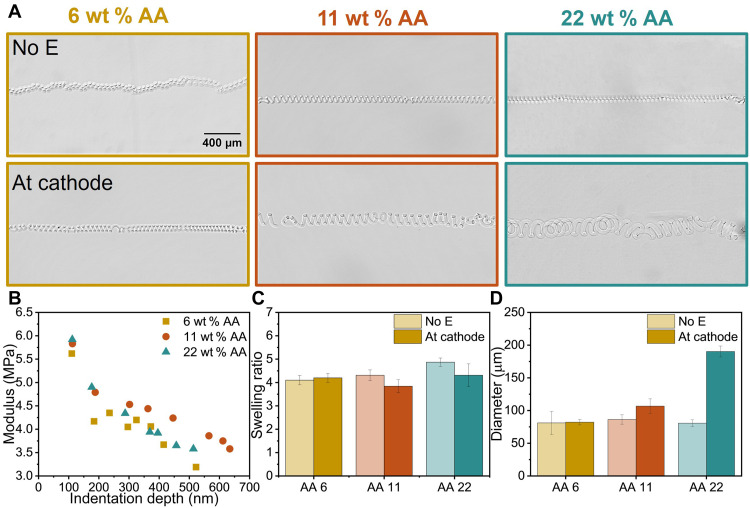
Size control of helix by tuning hydrogel compositions. (**A**) The microscope images of helical microfibers with different AA concentrations without electric fields and at the cathode. All the images had the same scale bar. (**B**) Depth-dependent elastic moduli of RIE modified dry microfibers measured by AFM indentation. Microfibers with different AA concentrations exhibited similar moduli due to the same cross-linker weight concentration. (**C**) The microfibers exhibited similar swelling ratios under cathode conditions compared to those observed without electric fields. (**D**) Before applying electric fields, microfibers coiled with similar helix diameters of microfibers as the microfibers had similar moduli and swelling ratios. When switching to the cathode, the helix diameters increased with increasing AA concentrations due to the stronger electrostatic repulsion between COO^−^ groups in AA 22 after proton migration. Consequently, this repulsion was anticipated to result in increased chain rigidity, leading to higher modulus and larger coiling diameters.

Beyond the temporal and spatial control over helix formation, the size of the helices was further shown to be controlled by the material composition and the electric field. Microfibers were fabricated with varying AA concentrations—6 wt % (AA 6), 11 wt % (AA 11), and 22 wt % (AA 22)—while maintaining a consistent cross-linker concentration (table S2). Upon initial release under the field-off condition, the microfibers with varying AA weight fractions showed similar coiling diameters, consistent with comparable mechanical properties and swelling ratios across different compositions. The widths of the swollen microfibers were nearly identical (Fig. 5C), and assuming isotropic swelling, the postswelling microfiber thickness (𝐷) was inferred to be comparable across all compositions, leading to a consistent pressure (Δπ) induced by differential swelling in the thickness direction. AFM indentation tests on RIE modified microfibers confirmed that elastic moduli (*E*) gradients remained invariant across different compositions (Fig. 5B), validated by the uniaxial tensile tests on swollen macroscopic hydrogels (fig. S14). Given similar swelling ratios and mechanical properties, the resulting coiling curvatures were comparable, leading to microfibers with similar helical diameters based on Eq. 1.

Upon applying the electric field and switching the ITO substrate with microfibers to the cathode, the microfibers exhibited swelling comparable to that in water when no electric field was applied (Fig. 5C) (*37*, *47*); however, the helix changed in size proportionally to AA concentration. We hypothesize that the hydrogel modulus increases in electric fields due to enhanced polymer chain alignment and organization. The electric field induced the dissociation of the close ion pairs formed between carboxylate groups and protons (─COOH). While the mobile protons (H^+^) migrated toward the cathode, the fixed carboxylate ions (─COO^−^) in the polymer network created electrostatic repulsion (*36*, *41*, *53*). This repulsion led to chain stiffening and reorganization in the lightly cross-linked networks, resulting in more ordered structures and increased hydrogel average modulus (*33*, *54*), which was consistent with measured larger coiling diameters in higher AA microfibers. Microfibers containing 22% AA, which had the highest concentration of acid groups, were anticipated to develop the most ordered polymer chains under electric fields. This was anticipated to result in the most notable average modulus increase and consequently the largest reduction in the coiling curvature, with helical diameters expanding to 2.3 times their initial size. AA11 microfibers showed a moderate increase in helical diameter from ~80 to ~105 μm. In contrast, AA6 microfibers, with their limited acid group content, exhibited negligible changes in swelling behavior and helical diameter under electric field stimulation (Fig. 5D). Moreover, the coiling and uncoiling kinetics accelerated with increasing AA concentration (fig. S15). Since both porosity and ionic concentration influence actuation speed and both vary with AA content, their contributions are coupled. While it is established that higher AA concentration enhances electroosmotic flow through increased charge density, the corresponding effect on porosity remained unclear. Direct characterization of microfiber porosity would be necessary, particularly because the NIL and RIE processing steps used in microfiber fabrication could alter pore structure in ways not reflected by macroscopic bulk hydrogels. Unfortunately, this characterization was not feasible because of the small scale of the microfiber. However, qualitative insight can be derived from the observed kinetic behavior. The consistent acceleration of actuation speed with AA concentration suggests that porosity was not substantially reduced at higher AA contents—if considerable pore closure had occurred, then it would have dominated over the ionic concentration effect and decelerated the kinetics, contrary to observations. These results establish a clear correlation between AA concentration and electric field–induced helix formation, enabling precise control over helix dimensions through composition tuning.

### Rotational actuation and microsphere manipulation

**Fig. 6.**
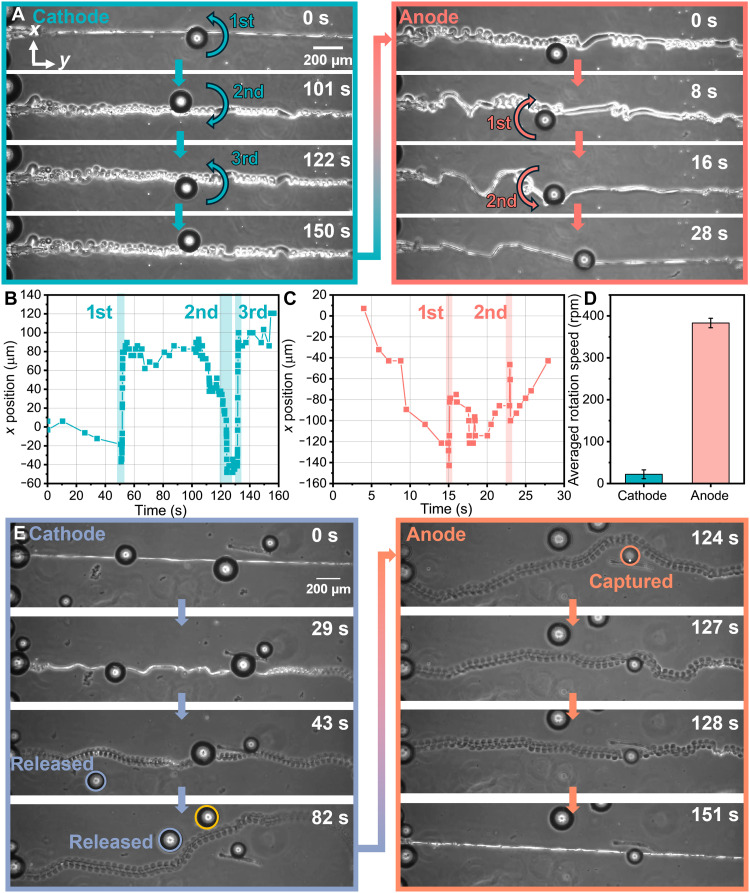
Rotational actuation and microsphere manipulation. (**A**) Sequential images of PMMA microsphere rotation through half-circles during microfiber coiling and uncoiling. All images share the same scale bar. (**B** and **C**) Tracked *x* position of the microsphere center during microfiber uncoiling and coiling, respectively. (**D**) Average rotation speed of the microsphere during microfiber coiling and uncoiling. The rotation speed under anode conditions was ~10 times faster than under cathode conditions due to the shorter rotational trajectory at the anode. (**E**) Sequential images of PMMA microspheres being released from actuated microfibers. All images share the same scale bar. PMMA microspheres were released under cathode conditions as the microfiber coiled because of reduced surface adhesion at the swollen microfiber surface, where the microsphere labeled with a yellow circle came from the flow. Conversely, under anode conditions, the microfiber uncoiled and moved to capture PMMA microspheres as surface adhesion increased for the deswollen microfiber.

In coiling and uncoiling, the microfibers produced rotary motion in response to electrical fields (movie S3), which was harnessed for rotary actuation by attaching a poly(methyl methacrylate) (PMMA) microsphere (radius, ~76.7 μm; mass, ~2.23 μg). Initially, the microsphere adhered to the microfiber in its straight configuration, which was at the bottom ITO substrate. Upon applying a 2-V potential to the ITO substrate with microfibers, configured as the cathode, the microfiber began to coil. Initially, coiling was observed in segments distal to the microsphere; however, as observed, the torque generated at this stage was insufficient to induce microsphere rotation. Rotation occurred only when the coiling front had advanced close enough (~1 mm) to the microsphere to transmit adequate torque. During this process, the microsphere first rotated upward and moved to the top of the microfiber. Subsequently, the microsphere rotated downward and upward, and completed three rotations in total, which were labeled as first, second, and third in Fig. 6A. The vertical (*x* axis) position of the microsphere was tracked during rotation (Fig. 6B). The rotation (π) was completed within 2 s at an average velocity of 22 rpm. When the field polarity was reversed, the microfiber uncoiled, and the microsphere completed two rotations at much higher speeds (~383 rpm). This acceleration occurred because the straightened microfiber had reduced dimensions and was no longer helical, requiring a shorter trajectory to complete the half-cycle rotation compared to the coiled configuration. Besides the polarity of the electric fields, the actuation speed can be further modulated by the magnitude of voltage based on the voltage-dependent coiling and uncoiling rates of microfibers (Fig. 2C). The demonstrated rotary actuation indicates the potential applications in microfluidic mixing and reconfigurable photonic displays.

In another set of experiments, we observed that the microspheres were controlled to be released and captured by the microfiber during coiling and uncoiling cycles (movie S4). AFM indentation tests revealed that adhesion at the deswelled microfiber surface was two orders of magnitude higher than at the swelled microfiber surface (fig. S16). Initially, two microspheres were attached to the deswelled microfiber (highlighted with purple circles in Fig. 6E). During coiling at the cathode, the microfiber swelled and surface adhesion decreased, causing the microspheres to be released. Upon switching to the anode, the microfiber uncoiled and simultaneously wiggled to capture another microsphere, which then adhered to the surface (highlighted with the pink circle in Fig. 6E). This controlled release and capture of microspheres demonstrates a potential pathway for sensor and payload delivery applications.

## DISCUSSION

Prior research has demonstrated the ability to replicate plant tendrils’ morphology through complex systems such as liquid crystalline elastomers (LCEs) with controlled orientation (*18*, *19*, *55*) or bilayer structures that require prestrain and interface control (*22*, *34*). Our work introduces a more accessible strategy using nanofabrication techniques to create helical structures that reproduce the fundamental differential strain mechanism in plant tendrils. Moreover, this principle is not limited to PAA hydrogels but can be broadly applied to any polyelectrolyte material under the same electric field configuration and two end-fixed boundary conditions, as the electro-osmotic mechanism is fundamentally independent of the specific ionic species. For instance, positively charged hydrogel microfibers (*56*, *57*) exhibit analogous swelling/deswelling behavior with reversed polarity—swelling occurs at the anode and deswelling at the cathode. Likewise, microfibers with strong ionic strength (*33*, *58*, *59*) display swelling and deswelling responses under applied electric fields, although with lower rates. Through controlled surface modification on hydrogel materials, our approach simplifies the biomimetic process while maintaining the essential mechanical principles that drive tendril winding.

Furthermore, this system enables the formation of helical structures at the mesoscopic scale (between microscopic and macroscopic dimensions). Previous demonstrations of winding actuation have predominantly remained at the macroscale due to inherent fabrication challenges. Current soft robotics approaches rely on two main methods: compliant systems and stimuli-responsive materials. Compliant systems, such as pneumatic (*60*) and hydraulic actuators (*61*), face fundamental limitations in miniaturization—the functional components cannot be feasibly scaled down and assembled at the microscale. In the stimuli-responsive systems, LCEs, the basis of ~90% of helical actuator systems, generate actuation through the phase transition of aligned liquid crystals (*10*, *18*, *19*, *52*, *55*, *62*). Achieving this alignment requires carefully controlled multistep programming, a process that becomes prohibitively difficult when scaling down to microscale dimensions. Apart from the temporal control, the spatial control of the helical structures is achieved with patterned substrates. This capability to dynamically and spatiotemporally control helical structures at the mesoscopic level creates opportunities for developing responsive small actuators and mini robots that can be activated precisely when and where needed.

In summary, this work demonstrates the biomimetic replication of tendril winding dynamics through controlled phase transitions in PAA-based hydrogels. By leveraging surface modifications achieved through RIE, we induced differential swelling patterns in PAA hydrogels that generate intrinsic curvature, which mimics the underlying tendril winding mechanism. The application of electric fields enables precise temporal and spatial control of helices through PAA hydrogel’s swelling/deswelling phase transition, which was further used for rotary actuation and microsphere release-capture. This system successfully mimics not only the dynamic development of plant tendril helices but also the underlying mechanisms of tendril coiling in natural systems. The ability to replicate and control these complex morphological transformations offers potential applications in smart material design.

## MATERIALS AND METHODS

### Fabrication of PAA microfibers

The microfibers were fabricated through the NIL and RIE process. The precursor mixture consisted of AA (Thermo Fisher Scientific, catalog no. 043359.K4) as the functional comonomer, 4-HBA (Thermo Fisher Scientific, catalog no. L11097.14) as a primary monomer, ethylene glycol methacrylate (MilliporeSigma) serving as the cross-linker, 2,2-dimethoxy-2-phenylacetophenone (MilliporeSigma) as the photoinitiator, and a trace amount of coumarin 153 dyes (MilliporeSigma) for fluorescent labeling. This formulated monomer mixture was first spin coated onto ITO glass substrates (KV-ITO-R10T07-2525-SK, Zhuhai Kaivo Optoelectronic Technology Co. Ltd.) that had been precoated with a polystyrene sodium sulfate (PSSS) (molecular weight, 70 kDa; Sigma-Aldrich, product no. 243051) sacrificial layer (~180 nm). The resulting polymer film was then subjected to NIL using a polydimethylsiloxane (PDMS) stamp (Dow 184 Sylgard silicone elastomer). The imprinting process was conducted in a Nanonex nanoimprinter (model NA-2608BA) under 15 psi and 25°C. In Nanonex, the monomer film was imprinted and simultaneously photopolymerized through 2-min ultraviolet (UV) light exposure to preserve the imprinted features. Following the imprinting and curing steps, the residual layer connecting the imprinted microfibers was removed through RIE. This etching process used oxygen plasma generated at 200-W radio frequency power with an oxygen gas flow rate of 50 standard cubic centimeters per minute and a duration of 4 min. The oxygen plasma effectively removed the residual polymer layer while converting the surface of the microfibers into brittle materials. This fabrication process yielded PAA hydrogel microfibers with a rectangular cross section, a thickness of ~8 μm, a width of ~10 μm, and lengths extending more than 76 mm. The rectangular cross section originated from the PDMS stamp prepared using a binary photolithographic silicon master. Notably, the cross-sectional shape—whether rectangular or circular—is not anticipated to significantly affect the coiling behavior of the microfibers. This is because one surface is always modified by RIE relative to the bottom surface in contact with the substrate. This asymmetry will persist regardless of cross-sectional geometry and will induce a gradient modulus, which drives microfiber differential swelling and coiling. With this said, cross-sectional shape differences may affect other aspects of the coiling morphology due to changes in the directional stiffness of the microfibers (*34*, *50*).

### Microscope imaging

Following fabrication, the 3-inch (7.62-cm) ITO glass wafer with the microfiber arrays was sectioned into smaller pieces for imaging (~2 cm by 2 cm). The two ends of ~5-mm sections of the microfibers were secured to the respective ITO substrates using a two-part epoxy adhesive. The prepared ITO glass with attached microfibers was then mounted onto the top of a custom 3D-printed frame (2 mm in thickness) with epoxy glue, with the bare ITO glass substrate glued at the bottom. This configuration created a complete electrical loop between the upper sample ITO and lower bare ITO electrodes while forming a chamber for subsequent microfiber release. Electrical grips were attached to both ITO glass pieces and connected to a dc power source, enabling controlled electrical stimulus. For microfiber release, Milli-Q water was introduced into the chamber. As the PSSS layer dissolved, the microfibers were released from the substrate, allowing them to respond to the electric fields. The bright-field and fluorescence imaging were conducted using a Zeiss Axio Observer Z1 automated microscope.

For imaging microfibers interacting with microspheres, the 3D-printed frame was first bonded to the ITO glass substrate containing the microfibers. Water was then added to the reservoir surrounded by the 3D-printed frame to release the microfibers. Subsequently, PMMA microspheres (Scientific Polymer Products Inc., catalog no. 037B) were introduced into the water reservoir. Upon gentle agitation, microspheres attached to the microfibers. After confirming microsphere attachment under the microscope, a bare ITO glass slide was bonded to the top of the frame to complete the electrical circuit. The remaining imaging procedures followed the same methods as experiments without microspheres.

### AFM indentation tests

AFM indentation tests were carried out for the modulus quantification. By leveraging highly sensitive tip-sample interactions in the *z* dimension, we simultaneously mapped the surface topography and quantified mechanical properties across a broad dynamic range (100 Pa to 100 GPa) at nanoscale resolution. Specifically, fast force mapping was used to rapidly generate high-resolution pixel arrays of force-distance curves using a Cypher ES system (Asylum Research). To ensure precise measurements of the PAA, a medium-soft probe (AC240TS-R3, Al-coated, spring constant = 2 N/m, resonance frequency = 70 kHz, tip radius = 7 nm, cantilever length = 240 μm) was selected. The elastic modulus was derived from the unloading curve using the Sneddon model, assuming a Poisson’s ratio of 0.5

$$\frac{dF_{N}}{d\delta }=\frac{16}{3\pi }E\delta {\left(\text{cotα}\right)}^{-1}$$

where $dF_{N}$ is the unloading slope, E is the Young’s modulus of PAA copolymer, δ is the indentation depth, and α is the half-angle of the AFM tip. The half-angle of the tip is ~36° after the wet anisotropic etching of Si{111} in NaOH-based solution for silicon bulk micromachining. For maximum pull-off force measurements, target forces of 20 and 10 nN were applied to deswelled and swelled PAA, respectively, at an approach velocity of 2 μm/s.

### Uniaxial tensile tests

Dogbone-shaped samples were fabricated in Teflon molds by photopolymerizing the monomer mixture under UV light. After curing, samples were equilibrated in water for 24 hours. The testing specimens had a length of ~20 mm, a width of ~6 mm, and a thickness of 3 mm. Uniaxial tensile tests were performed using a TA.XT Plus Texture Analyzer (Texture Technologies) with TA-96B grippers and a 5-kg load cell at a crosshead speed of 10 μm/s up to a target displacement of 0.2 mm. A small displacement was used to prevent both fracture of the swollen hydrogels during clamping and specimen slippage. Young’s modulus was determined from three independent samples, and data are presented as means ± SD.

## Supplementary Materials

### The PDF file includes:

Supplementary Text Figs. S1 to S16 Tables S1 and S2 Legends for movies S1 to S4 References
