# Supplementary material for: Bioinspired spatiotemporal control of microhelix formation and actuation
Source: Sci Adv. 2026 Jun 5;12(23):eaed9514. doi: 10.1126/sciadv.aed9514 (PMC13240167; doi:10.1126/sciadv.aed9514)
Supplement: Supplementary file 1 — Supplementary Text Figs. S1 to S16 Tables S1 and S2 Legends for movies S1 to S4 References [file sciadv.aed9514_sm.pdf]

Science Advances
AAAS

# Supplementary Materials for

## Bioinspired spatiotemporal control of microhelix formation and actuation

Xin Hu *et al.*

Corresponding author: Alfred J. Crosby, acrosby@umass.edu

*Sci. Adv.* **12**, eaed9514 (2026)
DOI: 10.1126/sciadv.aed9514

**The PDF file includes:**

Supplementary Text
Tables S1 and S2
Figs. S1 to S16
Legends for movies S1 to S4
References

**Other Supplementary Material for this manuscript includes the following:**

Movies S1 to S4

## Supplementary Text

Simulation of the microfiber coiling configuration

In our experiments on spontaneously coiling microfibers, the two ends of each strand are held fixed to the substrate with glue, which locally restricts their ability to rearrange. We consider these boundary conditions as *"fixed grips"*; that is, the ends of each microfiber can neither translate nor rotate. Despite the fact that the microfiber is mathematically an *open curve* (the ends are not connected to form a closed loop), these boundary conditions effectively force the loop into a closed configuration, which is therefore subject to the topological constraints of closed curves. The most important implication of these boundary conditions for the present study is that the Linking Number ($Lk$) of the microfiber must be conserved. For a single microfiber, the Linking Number can be expressed as

$$Lk = Wr + Tw \tag{1}$$

where $Wr$ is the Writhe of the microfiber (which is crudely a measure of the helicity) and $Tw$ is Twist (which measures how much the material frame of the microfiber rotates around its central "backbone" curve). For more detailed mathematics, we refer the reader to Berger (*63*) and Fuller (*64*). As such, the microfibers in experiments are free to change their configuration via changes in $Wr$ or $Tw$ in response to the applied field (and resulting deformations in the material), subject to the constraint that $Wr + Tw = const$. Importantly, in the present study, the initial configuration is a straight, untwisted microfiber (that is, $Wr_0 = 0$, $Tw_0 = 0$), so any configuration must satisfy $Wr = -Tw$. In our experiments, microfibers adopt configurations with regions of positive $Wr$ (right-handed helicity) and regions with negative $Wr$ (left-handed helicity), separated by small regions of perversion (with both positive and negative $Tw$), such that the net $Wr$ cancels along the length (and thereby satisfying the constraint $Lk = 0$). Therefore, the coexistence of different chirality within a microfiber (coiled from a straight, untwisted configuration) is driven primarily by the boundary conditions enforced by topology.

| | Experiment | Simulation |
|---|---|---|
| t = 0 | 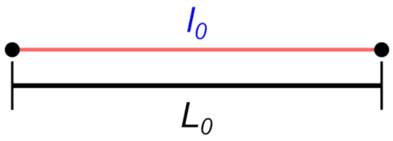 $l_0$, $L_0$ | 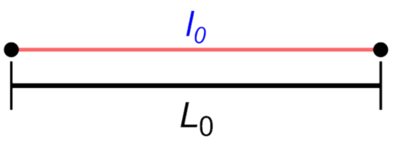 $l_0$, $L_0$ |
| t = ∞ | 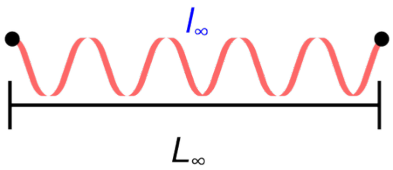 $l_\infty$, $L_\infty$ | 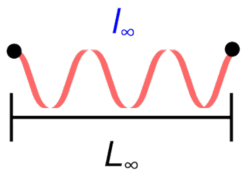 $l_\infty$, $L_\infty$ |
| conditions | $L_0 = L_\infty = l_0$<br>$\Phi^* l_0 = l_\infty$<br>$l_\infty = \Phi^* L_0$ | $L_0 = l_\infty = l_0$<br>$\Phi^* L_\infty = L_0$<br>$l_\infty = \Phi^* L_0$ |

**Table S1**. The comparisons between experiment and simulation parameters. In experiments, the two ends of the microfibers were fixed and the microfibers coiled into helices due to swelling. In

simulations, the ends of the microfibers were gradually brought closer to simulate the extension of swelling in the experiments.

Discrete Kirchhoff Rod Simulations

To demonstrate the consequences of the topological constraint $Lk = 0$, we conduct simulations based on Kirchhoff Rod Theory, where the elastic energy of a microfiber is given by

$$\varepsilon = \frac{1}{2}\int ds\left[B_1(\kappa_1(s) - \kappa_0)^2 + B_1(\kappa_2(s))^2 + C(\omega(s))^2\right] \quad (2)$$

The integral in Eqn. (1) runs along the arclength of the microfiber ($s$) from 0 to $L$ and the intrinsic (stress-free) curvature driven by the differential swelling is $\kappa_0$. The deformed configuration is described by curvatures in two principal directions, $\kappa_1(s)$, $\kappa_2(s)$, and the twist of the rod $\omega(s)$, and $B_1$, $B_2$, and $C$ are the respective bending and twisting moduli. Owing to the separation of scales, that is $t, w \ll L$ (where $t$ and $w$ are the cross-sectional thickness and width and $L$ is the length), we treat our microfibers as inextensible and unshearable. In other words, the lowest energy terms in the energy only penalize bending (in two principle directions) and twisting, as is common in the mechanics of rods (*65*). In general, the bending and twisting moduli are functions of both the material properties and geometry of the cross-section, where $B_i = EI_i$ and $C = \mu J$ (and $E$ is the Young's Modulus, $I_i$ is the principle moment of inertia in the $i$ direction, $\mu$ is the shear modulus $\mu = E/2(1+\nu)$ where $\nu$ is the poisson ratio, and $J$ is the torsional rigidity of the cross-section shape). For the rectangular prismatic microfibers studied in our experiments with thickness $t$ and $w$, $B_{1,rect} = E\frac{wt^3}{12}$, $B_{2,rect} = E\frac{tw^3}{12}$, and $C_{rect} \simeq E\frac{0.196wt^3}{2(1+\nu)}$ (*66*). For simplicity, we instead model our cross-sections as isotropic (i.e. circular, where we assume $w \sim t \sim D$, where $D$ is the cross-section diameter corresponding to an disk of equal area to our rectangular section, $D = 2\sqrt{wt/\pi}$). In this case, $B_1 = B_2 = E\frac{\pi D^4}{64}$, and $C = E\frac{\pi D^4}{64(1+\nu)}$. In our simulations, we assume incompressibility of the material ($\nu = ½$), yielding $B_1/B_2 = 1$ and $C/B_1 = 2/3$. Comparing our experimental geometries to simulations, we have $B_{1,rect}/B_1 \simeq 0.73$, $B_{2,rect}/B_2 \simeq 1.49$, and $C_{rect}/C \simeq 0.86$; that is, intuitively, approximating our rectangular cross-section as a circle (with the same cross-sectional area) slightly overestimates the bending stiffness in the thin direction and twisting rigidity and slightly underestimates the bending stiffness in the thick direction. However, these parameters are sufficient to demonstrate the role of the topological constraints described above.

Simulations are conducted using a discretized version of Eqn. (2) in LAMMPS developed by Brackley (*67*) and based on code originally implemented by Lequieu (*68*), where the shape bending deformations are described by bond angles and twisting deformations are described by dihedral angles. Here, the strand is discretized into $N$ total beads with arclength $L$. We set the preferred bond angles (derived from the continuum theory $\kappa_o$) and prevent self-intersections of the microfiber through a purely repulsive Weeks-Chandler-Anderson potential (with cutoff distance given by the microfiber diameter, $D$). Notably, even though the mechanics and excluded volume represent a microfiber with circular cross-sections, we render the structures as prismatic beams to display the twist of the material.

We model the glued ends in our experiments by restricting the translational and rotational degrees of freedom of the ends of each strand in simulations (discretized bead $n = 1$ and $n = N$). All other beads are free to translate and rotate, subject to their bonding constraints. In the simulations which correspond to our experiments (Fig. 1), the initial configuration of the rod is straight and untwisted ($Lk = 0$). To model the linear swelling of the material, we quasistatically decrease the distance ($L_0$) between the fixed grips ($n = 1$ and $n = N$), running overdamped Langevin dynamics at each step in the end shortening procedure, which effectively increases the ratio of microfiber length $L$ to end-to-end distance $L_0$, allowing the strand to coil without stretching. Note that this procedure is conceptually analogous to the experiments, in which $L_0$ is held constant and $L$ increases due to the swelling of the microfiber. In simulations, the ratio $L/L_0$ is continuously increased until it reaches length ratio corresponding to fully swollen experimental systems.

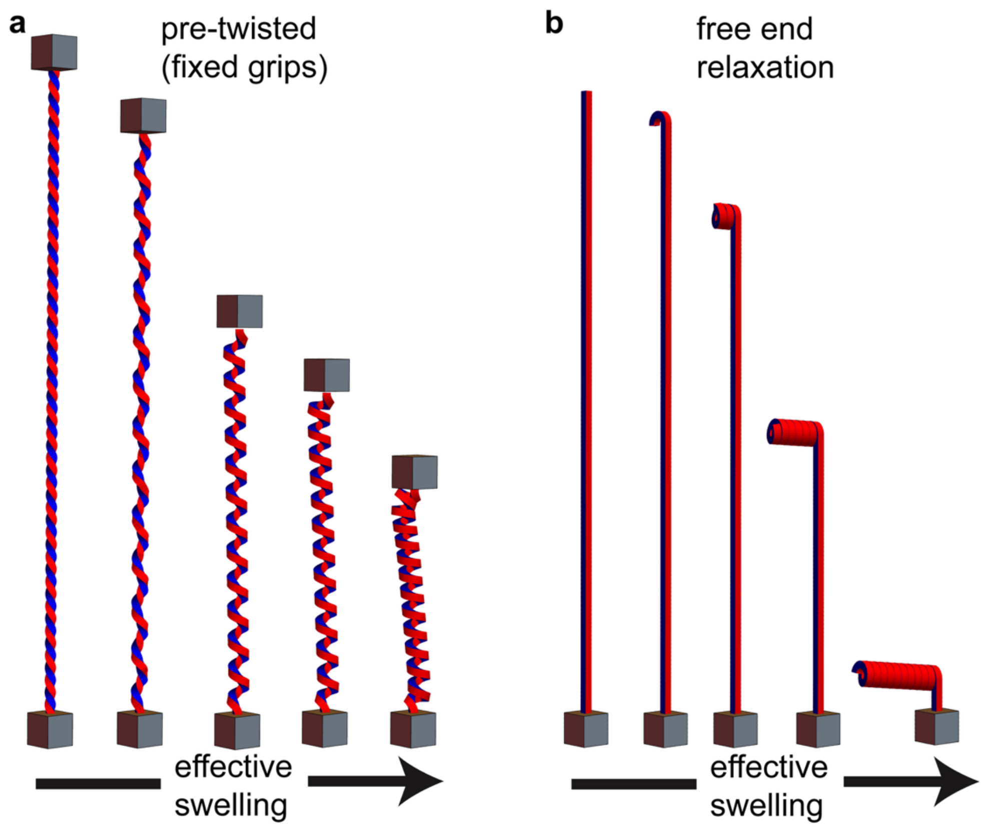


**Fig. S1.** Approaches to generate perversion-free helices (with $\mathbf{L_0} = \mathbf{80w}$ and $\boldsymbol{\kappa_0} = (\mathbf{2w})^{-\mathbf{1}}$. (a) Filament held in fixed grips is pre-twisted ($\mathbf{Tw_o} \neq \mathbf{0}$); as the ends are brought closer together, twist is exchanged for writhe. (b) Filament is held in fixed grips at one end while the other end is free; and as such, the link is not fixed.

To demonstrate the interplay between topology and morphology, we also consider two additional scenarios. In the first, we pre-twist the microfiber such that $|Tw_0| > 0$ (therefore constraining $|Lk| > 0$); as predicted via topology, running the same end-shortening procedure now yields coiled microfibers with a net writhe $|Wr| > 0$. If the initial twist is specified appropriately (that is $|Tw_0| = |Wr|$ where $|Wr|$ corresponds to the target swollen helical configuration), it is

possible to form helices with only one handedness. Here, the net chirality is *inherited* from the initial twist (Fig. S.1a). In the second scenario, we restrain only one end of the microfiber, allowing the $n = N$ bead to also translate and rotate (analogous to one end becoming detached from the substrate). In this case, $Lk$ is no longer fixed and can evolve during the relaxation (that is $dLk/dt \neq 0$). Assuming the relaxation propagates sufficiently slowly from the free end, $|Wr|$ can increase (via rotation of the free end), again yielding configurations with only one handedness (Fig. S.1b). To model this in simulations, we sequentially relax increasingly larger segments starting from the free end (which is conceptually analogous to the microfiber slowly peeling off the substrate, where this peeling front propagates down the length from the free end). Together, these examples highlight how the existence of multiple chiralities separated by perversions (chirality inversions) is driven by the boundary conditions in our experiments. Code to conduct each of these three relaxation procedures and visualize the output is available at (https://scholarworks.umass.edu/entities/publication/c46df667-34a9-47ab-94d6-ae17fe519c1a).

COMSOL simulation

COMSOL Multiphysics 5.1 was used to simulate the electric potential distribution in the setup with patterned ITO substrates. An electrostatic model was constructed with the following configuration: the bottom substrate consisted of uniform ITO (75 mm wide), while the top substrate featured a patterned design with ITO on the left half (2.5 mm wide) and glass on the right half (2.5 mm wide). Both materials were assigned a relative permittivity of 6. The gap between the two substrates (2 mm) was filled with water, which has a relative permittivity of 80. The boundary conditions were set with the top ITO electrode at 2 V and the bottom ITO electrode at 0 V (ground). The entire model was enclosed within a large circular air domain to define the outer boundary.

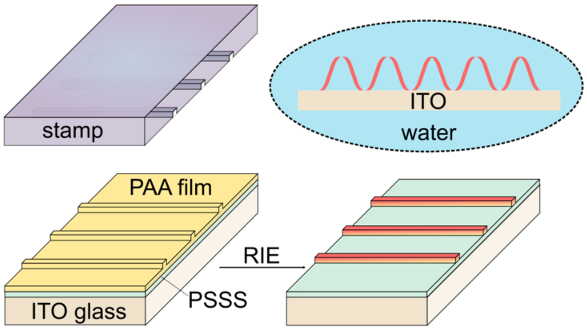


**Fig. S2. The reactive ion etching (RIE) process for microfiber fabrication.** RIE removed the residual layer connecting the microfibers and simultaneously modified the top surfaces of the microfibers, which contributed to the differential swelling in coiling.

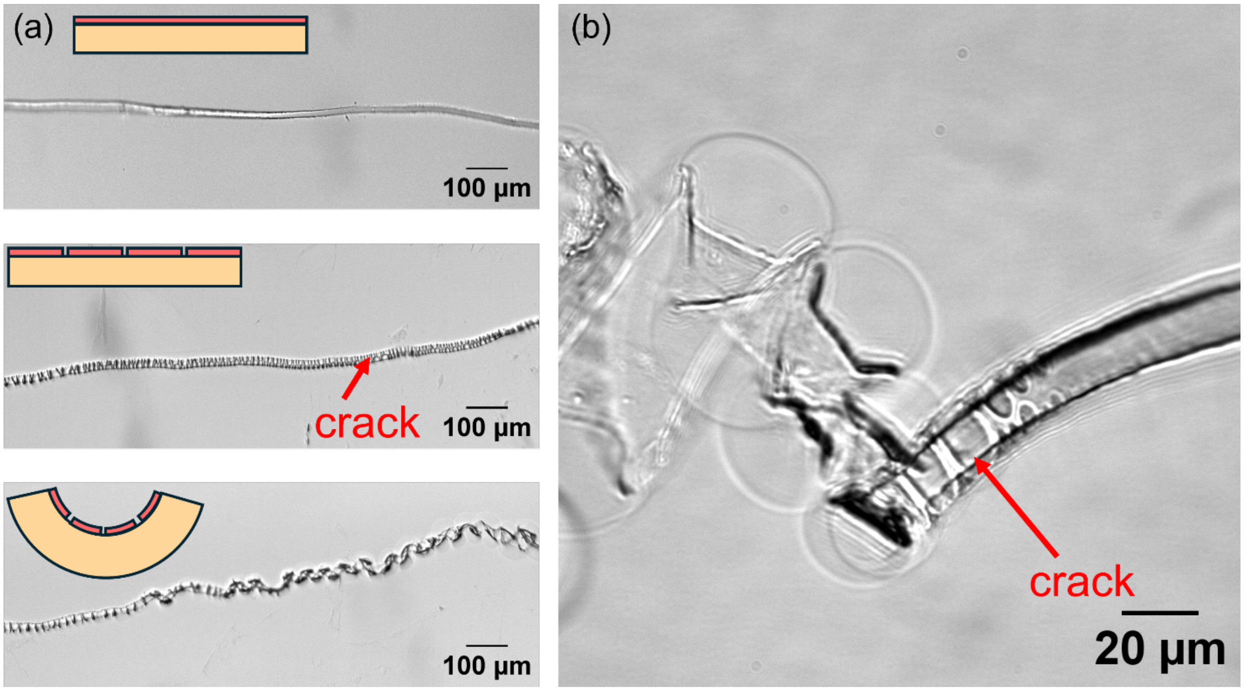


**Fig. S3. The fracture of the microfiber top surface during swelling.** (a) Progressive swelling of the microfiber with cracks. The crack formed during initial swelling (middle image) and spread out in the coiling. All the images had the same scale bar. (b) The zoomed-in image showed the crack morphology.

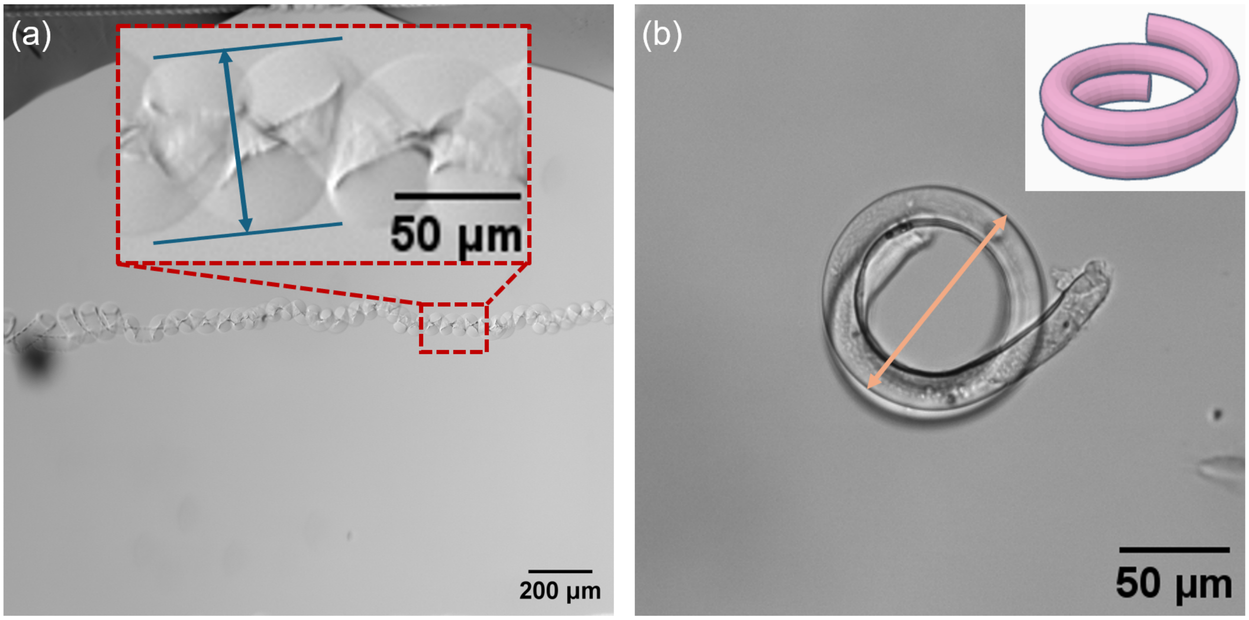


**Fig. S4. Coiling behavior of microfibers in water.** Images were acquired using an optical microscope in transmission mode as the microfibers underwent swelling in water in the absence of an applied electric field. (a) The coiling diameter of the long microfiber, with both ends fixed, was measured at ~79 ± 6.28 μm, determined from the edge-to-edge distance of the microhelices as indicated by the double arrow. (b) A short microfiber cut by the razor blade with both ends free, exhibiting an average coiling diameter of ~90 ± 5.60 μm, measured from 3 segments across 3 independent microfibers. The comparable coiling diameters under these two boundary conditions suggest that boundary conditions do not significantly influence microfiber coiling.

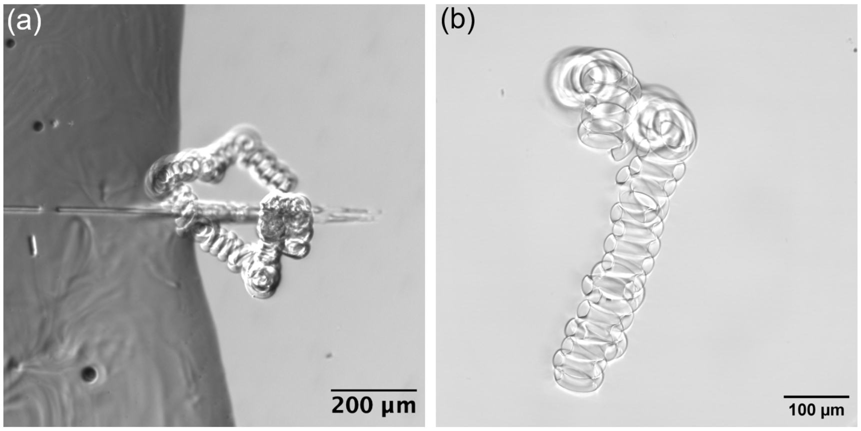


**Fig. S5. The image of the microfiber with (a) one end free and (b) two ends free.** Due to gravity, the coiled microfiber formed a blob in 3D space, which impeded the observation of the microfiber configuration.

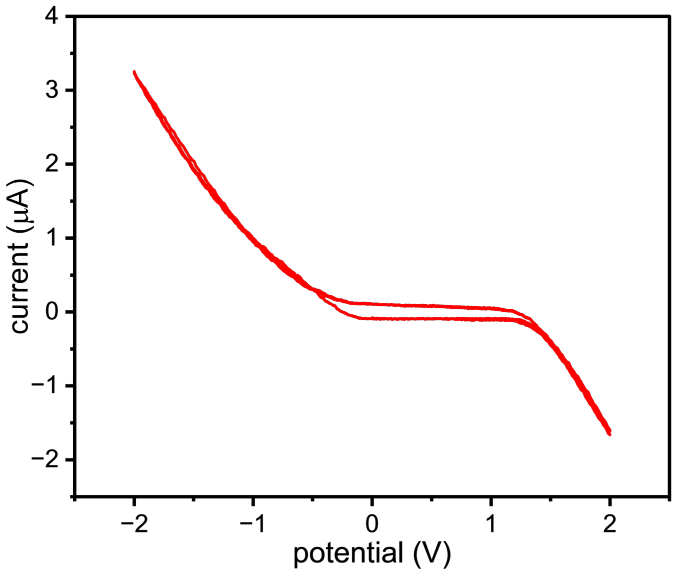


**Fig. S6. The cyclic voltammetry result of Milli-Q water.** The scan rate: 100 mV/sec; electrodes: ITO glass; reference electrode: Ag/AgCl. The redox peaks appeared in the CV measurements, which indicated electrolysis of water.

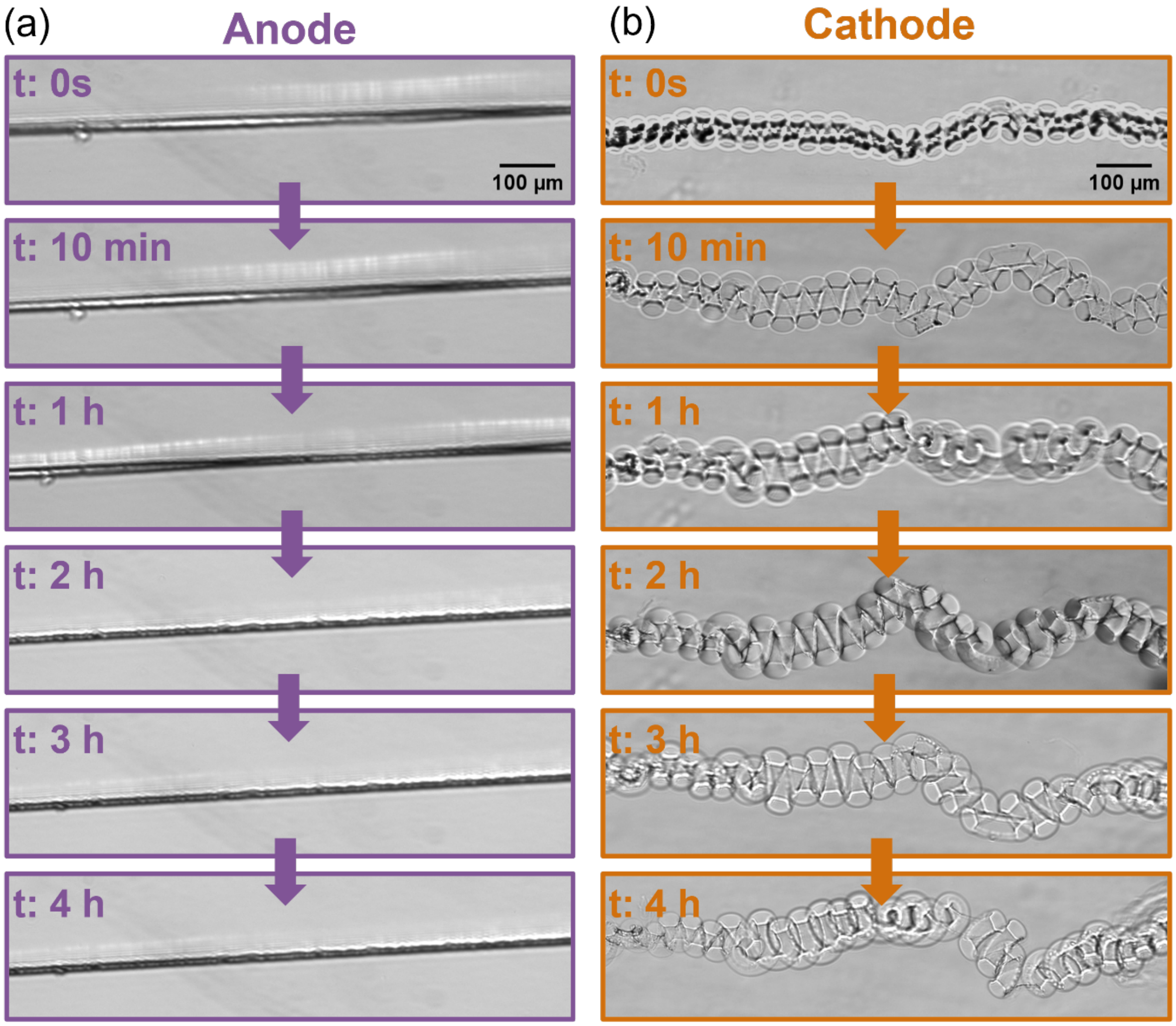


**Fig. S7. Long-term stability of microfibers under sustained electric field polarity.** Microfibers exhibited the persistent straight and coiled configurations when maintained near the anode (a) and cathode (b) for four hours without observable morphological alterations.

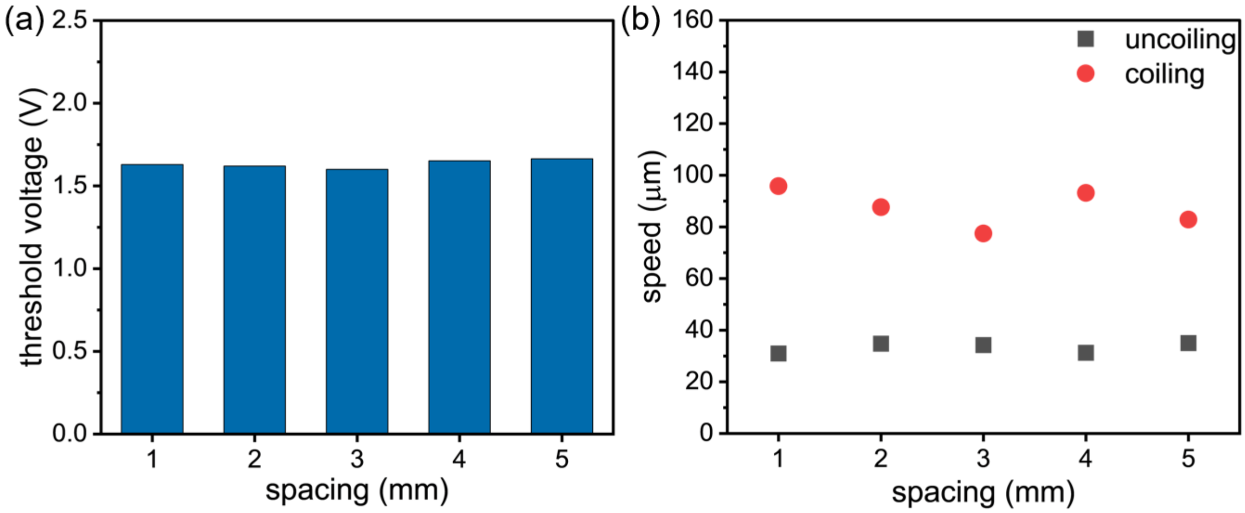


**Fig. S8. The threshold voltage and coiling/uncoiling kinetics at different electrode spacings.** (a) The threshold voltage remained consistent across varying electrode spacings, stabilizing at approximately 1.6 V, indicating that this parameter is independent of electrode spacing. (b) With the input voltage held constant at approximately 1.7 V across all electrode spacings, the coiling/uncoiling kinetics remained relatively independent of the electrode spacing.

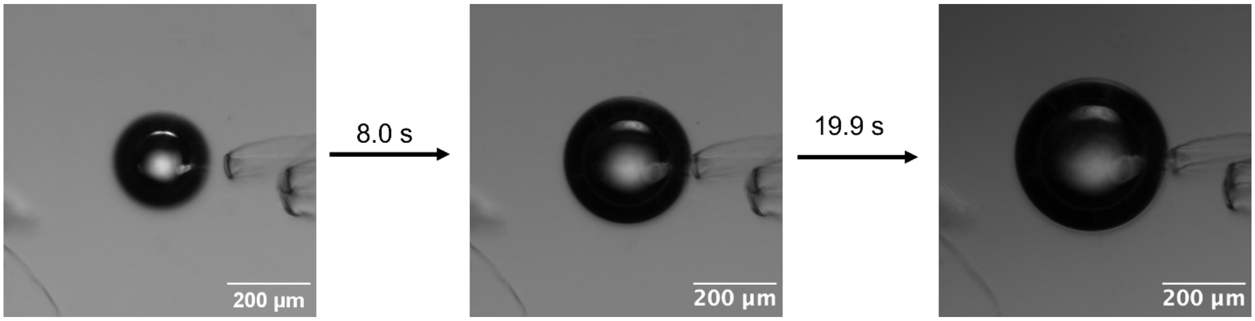


**Fig. S9. Bubble formation due to the hydrolysis of water.** Under an applied voltage of 5 V, bubbles nucleated at the ITO glass surface and expanded over time. All the images had the same scale bar.

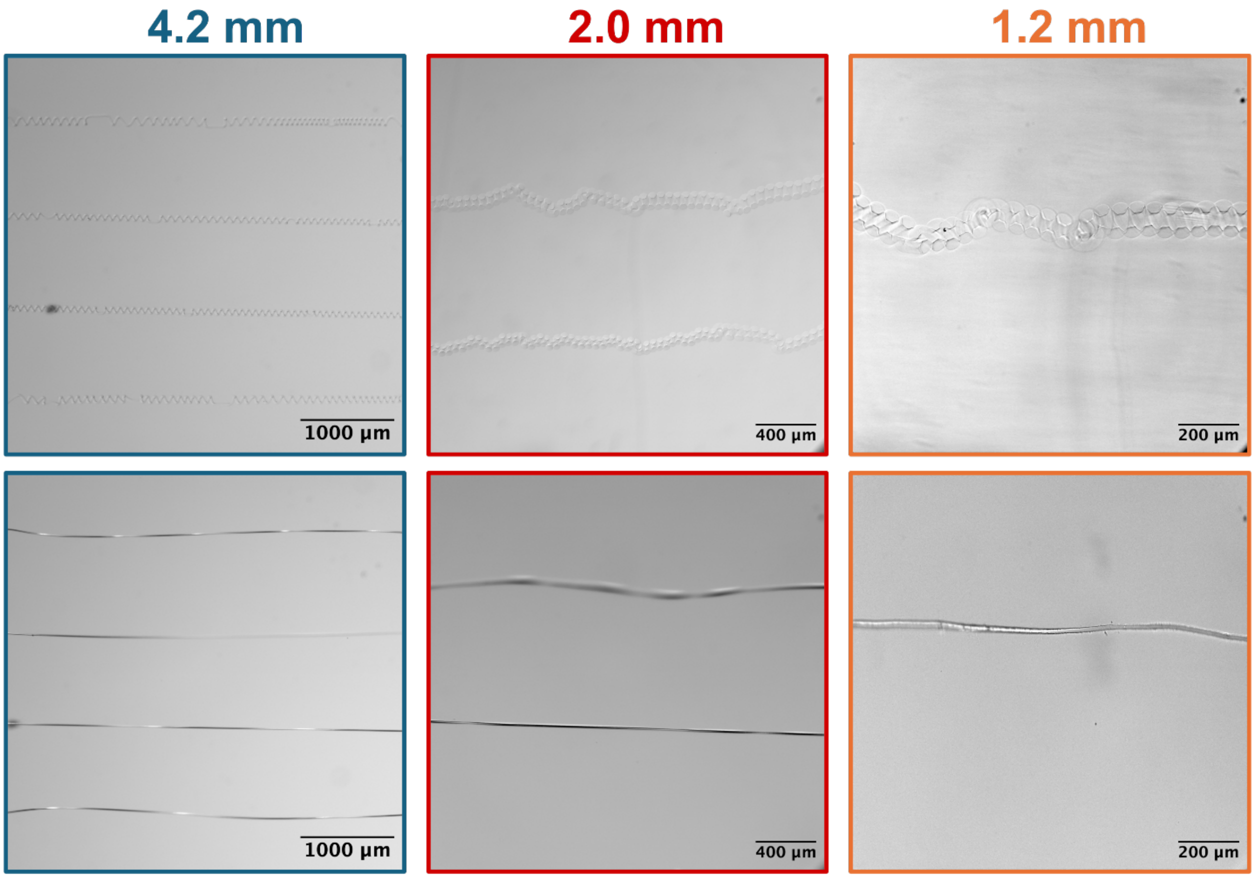

**Fig. S10. The coiling and uncoiling of microfibers with different lengths.** Microfibers with three different lengths were actuated to transition between coiling and uncoiling states, which demonstrated that microfiber lengths had no impact on the actuation.

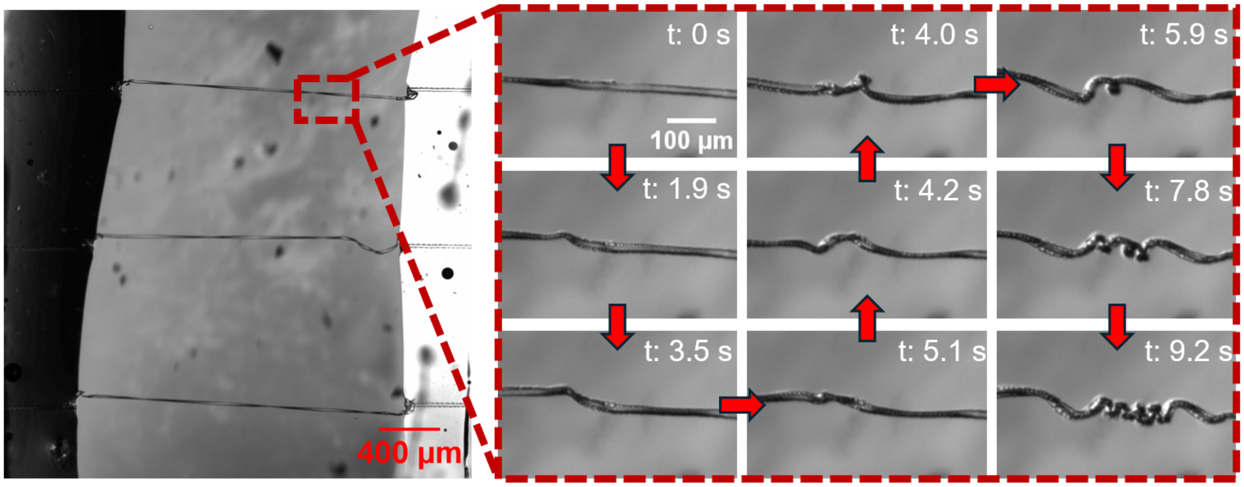


**Fig. S11. Initial coiling of a microfiber at the cathode.** Low-magnification imaging was employed to locate the initial coiling segment, and time-lapse zoom-in images were acquired to capture its evolution. At the onset of coiling, the segment underwent bending accompanied by rotation of the entire microfiber. Following the formation of the first coil, this rotational behavior propagated further along the fiber.

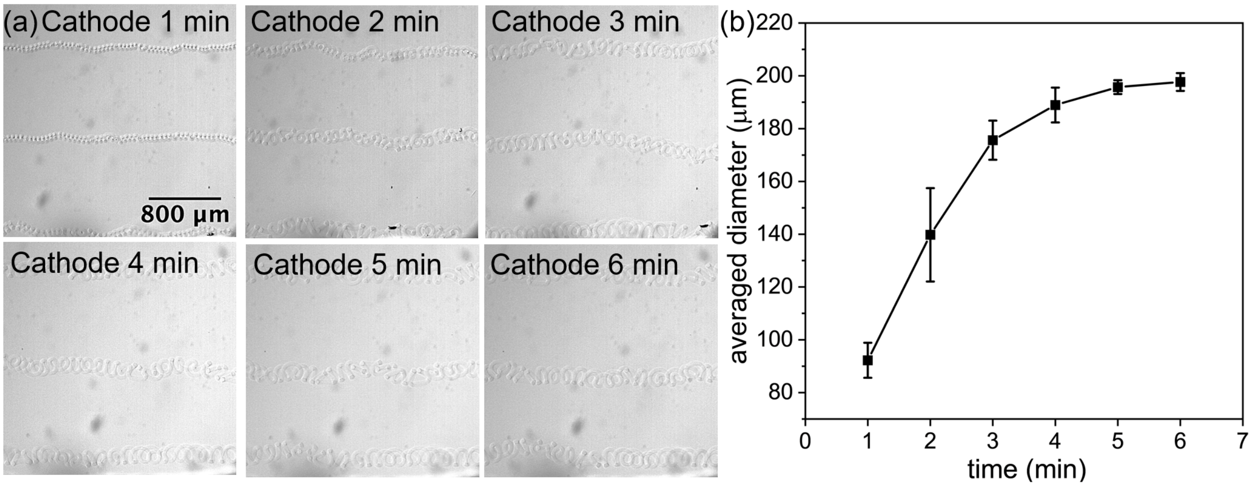


**Fig. S12. The swelling of the microfiber after coiling.** (a) Sequential microscope images of AA 22 microfibers undergoing swelling after coiling transformation for 6 min. All the images had the same scale bar. (b) Temporal evolution of average coil diameter. Following complete coil formation, the microfiber continued to swell, increasing coil dimensions until reaching equilibrium after 5 min.

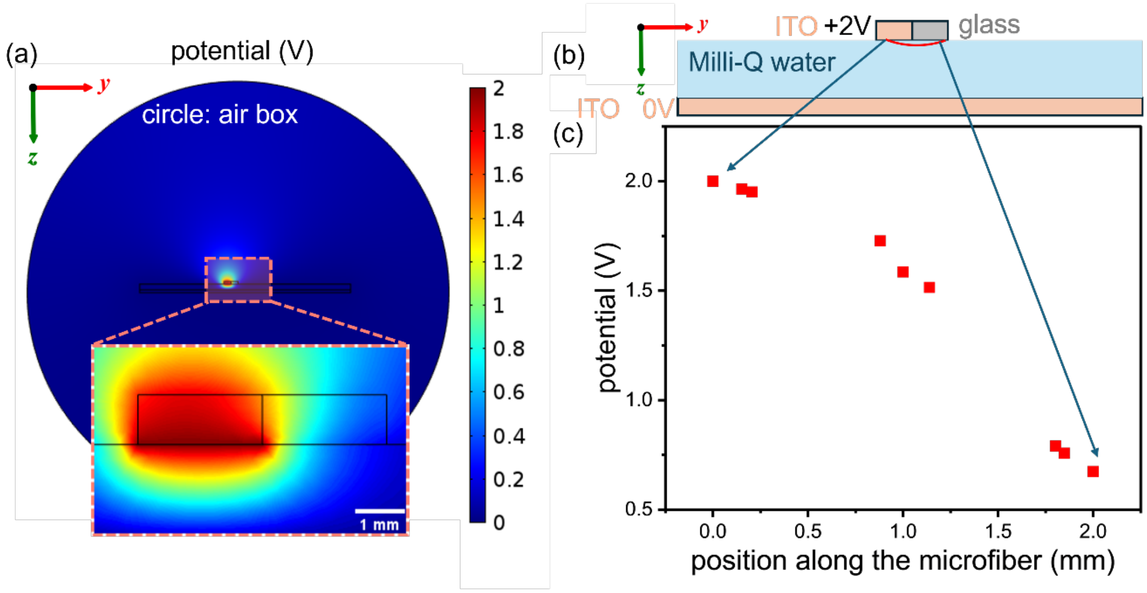


**Fig. S13. COMSOL simulation of the potential distribution with patterned substrates.** (a) The 2D maps of the electric potential distribution at the cross-section of the electric setup. (b) Schematic of the simulation configuration. (c) Representative electric potential gradient along a loop down a 2-mm microfiber across the ITO/glass boundary, which was decreasing from one end to another.

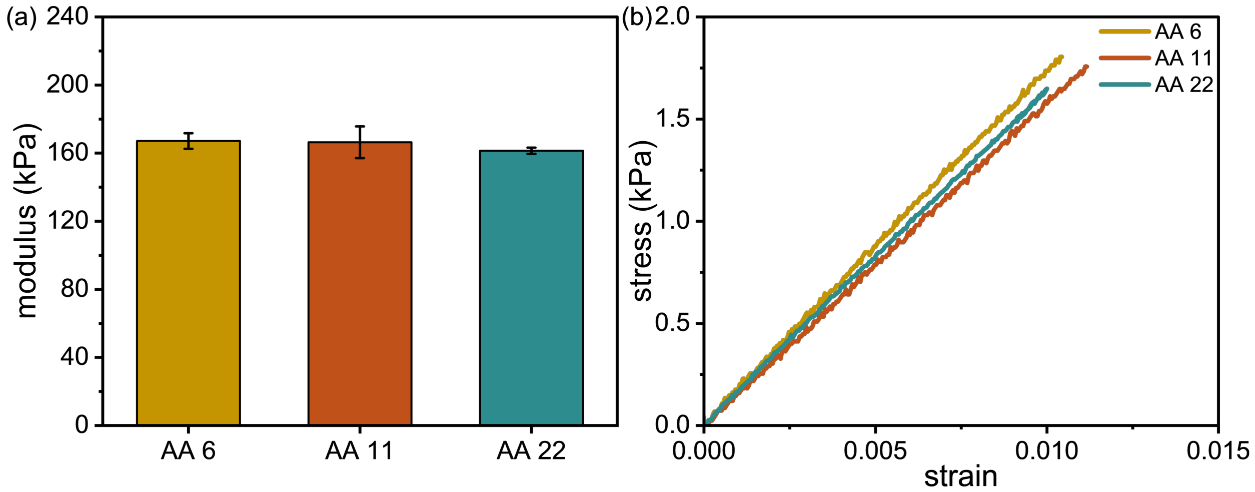


**Fig. S14. Modulus of swollen macroscopic hydrogel from uniaxial tensile tests.** (a) The average modulus of macroscopic swollen hydrogels from three independent samples. (b) Representative stress-strain curves in the uniaxial tensile tests.

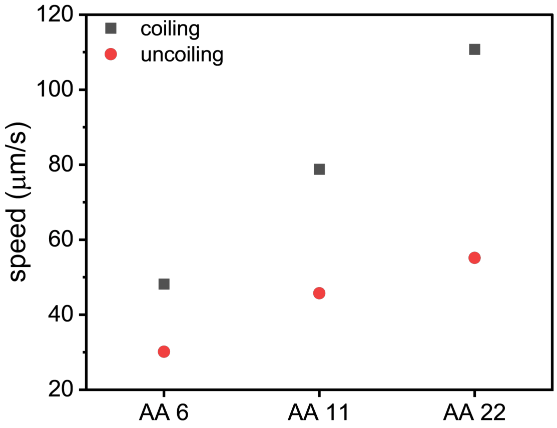


**Fig. S15. Coiling and uncoiling speeds of microfibers with different AA concentrations.** At higher AA concentrations, the microfibers exhibited faster actuation, which is attributed to ion-concentration-dependent electroosmotic flow.

(a)

| Max pull-off force (nN) | Average | St. Dev. |
|---|---|---|
| Deswelled | 35.49 | 6.96 |
| Swelled | 0.67 | 0.30 |

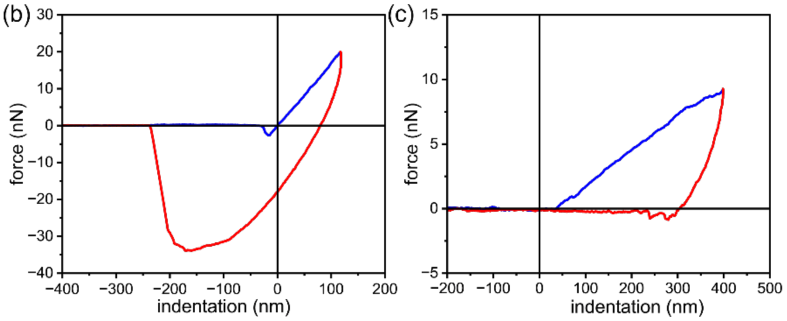


**Fig. S16.** The average and standard deviation of max pull-off forces for the deswelled and swelled microfibers measured by the AFM indentation tests, which indicated that the adhesion at the deswelled microfiber surfaces was two orders of magnitude higher than the swelled microfiber surfaces. (b) and (c) are the force-indentation data for the deswelled and swelled microfiber, respectively.

| Sample Name | AA (wt.%) | 4-HBA (wt.%) | CL (EGDMA) (wt.%) | PI (DMPA) (wt.%) |
|---|---|---|---|---|
| AA 6 | 6 | 90 | 1 | 3 |
| AA 11 | 11 | 85 | 1 | 3 |
| AA 22 | 22 | 74 | 1 | 3 |

**Table S2.** The material compositions of microfibers with different AA concentrations.

**Movie S1.** Video showing the continuous uncoiling and coiling of the microfibers as the field polarity switches, played at 3× speed.

**Movie S2.** Video showing the uncoiling of the microfibers in the ITO regions at the anode, played at 16× speed.

**Movie S3.** Video showing rotary actuation of a PMMA microsphere attached to the microfiber as it coiled at the cathode and uncoiled at the anode (10x fast).

**Movie S4.** Video showing PMMA microspheres being released at the cathode during microfiber coiling and recaptured at the anode during uncoiling (10x fast).

## REFERENCES

1. A. Imberty, H. Chanzy, S. Pérez, A. Bulèon, V. Tran, The double-helical nature of the crystalline part of A-starch. *J. Mol. Biol.* **201**, 365–378 (1988).

2. C. W. Lloyd, Toward a dynamic helical model for the influence of microtubules on wall patterns in plants. *Int. Rev. Cytol.* **86**, 1–51 (1984).

3. M. Schilthuizen, A. Davison, The convoluted evolution of snail chirality. *Naturwissenschaften* **92**, 504–515 (2005).

4. L. Tombolato, E. E. Novitskaya, P. Y. Chen, F. A. Sheppard, J. McKittrick, Microstructure, elastic properties and deformation mechanisms of horn keratin. *Acta Biomater.* **6**, 319–330 (2010).

5. J. D. Watson, F. H. C. Crick, Molecular structure of nucleic acids: A structure for deoxyribose nucleic acid. *Nature* **171**, 737–738 (1953).

6. R. M. Macnab, Bacterial flagella rotating in bundles: A study in helical geometry. *Proc. Natl. Acad. Sci. U.S.A.* **74**, 221–225 (1977).

7. A. Goriely, M. Tabor, Spontaneous helix hand reversal and tendril perversion in climbing plants. *Phys. Rev. Lett.* **80**, 1564–1567 (1998).

8. T. McMillen, A. Goriely, Tendril perversion in intrinsically curved rods. *J. Nonlinear Sci.* **12**, 241–281 (2002).

9. J. S. Wang, G. Wang, X. Q. Feng, T. Kitamura, Y. L. Kang, S. W. Yu, Q. H. Qin, Hierarchical chirality transfer in the growth of towel gourd tendrils. *Sci. Rep.* **3**, 3102 (2013).

10. M. Wang, B.-P. Lin, H. Yang, A plant tendril mimic soft actuator with phototunable bending and chiral twisting motion modes. *Nat. Commun.* **7**, 13981–13988 (2016).

11. M. H. Godinho, J. P. Canejo, G. Feio, E. M. Terentjev, Self-winding of helices in plant tendrils and cellulose liquid crystal fibers. *Soft Matter* **6**, 5965–5970 (2010).

12. C. Darwin, *The Movements and Habits of Climbing Plants* (John Murray, 1876); www.google.com/books/edition/The_Movements_and_Habits_of_Climbing_Pla/jhEAAAAAQAAJ?hl=en&gbpv=0.

13. M. J. Jaffe, A. W. Galston, The physiology of tendrils. *Annu. Rev. Plant Biol.* **19**, 417–434 (1968).

14. D. Nahar, P. M. Yanik, I. D. Walker, "Robot tendrils: Long, thin continuum robots for inspection in space operations" in *2017 IEEE Aerospace Conference Proceedings* (IEEE, 2017), pp. 1–8.

15. I. Must, E. Sinibaldi, B. Mazzolai, A variable-stiffness tendril-like soft robot based on reversible osmotic actuation. *Nat. Commun.* **10**, 344 (2019).

16. F. Meder, S. P. M. Babu, B. Mazzolai, A plant tendril-like soft robot that grasps and anchors by exploiting its material arrangement. *IEEE Robot. Autom. Lett.* **7**, 5191–5197 (2022).

17. M. Farhan, F. Klimm, M. Thielen, A. Rešetič, A. Bastola, M. Behl, T. Speck, A. Lendlein, Artificial tendrils mimicking plant movements by mismatching modulus and strain in core and shell. *Adv. Mater.* **35**, e2211902 (2023).

18. Z. Hu, Y. Li, J. Lv, Phototunable self-oscillating system driven by a self-winding fiber actuator. *Nat. Commun.* **12**, 3211 (2021).

19. C. Zhang, G. Fei, X. Lu, H. Xia, Y. Zhao, Liquid crystal elastomer artificial tendrils with asymmetric core–sheath structure showing evolutionary biomimetic locomotion. *Adv. Mater.* **36**, 2307210 (2024).

20. C. G. Meloche, J. P. Knox, K. C. Vaughn, A cortical band of gelatinous fibers causes the coiling of redvine tendrils: A model based upon cytochemical and immunocytochemical studies. *Planta* **225**, 485–498 (2006).

21. A. J. Bowling, K. C. Vaughn, Gelatinous fibers are widespread in coiling tendrils and twining vines. *Am. J. Bot.* **96**, 719–727 (2009).

22. S. J. Gerbode, J. R. Puzey, A. G. McCormick, L. Mahadevan, How the cucumber tendril coils and overwinds. *Science* **337**, 1087–1091 (2012).

23. E. Jabbari, M. E. Khosroshahi, M. Boroujerdi, Swelling characterization of anionic acrylic acid hydrogel in an external electric field. *Iranian Polymer Journal* **15**, 891–900 (2006).

24. B. D. Johnson, D. J. Beebe, W. C. Crone, Effects of swelling on the mechanical properties of a pH-sensitive hydrogel for use in microfluidic devices. *Mater. Sci. Eng. C* **24**, 575–581 (2004).

25. K. S. De, N. R. Aluru, B. Johnson, W. C. Crone, D. J. Beebe, J. Moore, Equilibrium swelling and kinetics of pH-responsive hydrogels: Models, experiments, and simulations. *J. Microelectromech. Syst.* **11**, 544–555 (2002).

26. G. H. Kwon, Y. Y. Choi, J. Y. Park, D. H. Woo, K. B. Lee, J. H. Kim, S. H. Lee, Electrically-driven hydrogel actuators in microfluidic channels: Fabrication, characterization, and biological application. *Lab Chip* **10**, 1604–1610 (2010).

27. G. Han Kwon, J. Yull Park, J. Yoon Kim, M. L. Frisk, D. J. Beebe, S.-H. Lee, S. Lee, G. H. Kwon, J. Y. Park, J. Y. Kim, M. L. Frisk, D. J. Beebe, Biomimetic soft multifunctional miniature aquabots. *Small* **4**, 2148–2153 (2008).

28. X. Hu, V. J. Einck, E. Chia, J. N. Pagaduan, D. E. Moed, T. Emrick, R. Katsumata, S. S. Nonnenmann, J. J. Watkins, A. J. Crosby, Reversible self-coiling of microfibers with tailored surfaces via elastocapillarity. *Small* **21**, e07151 (2025).

29. G. S. Oehrlein, R. J. Phaneuf, D. B. Graves, Plasma-polymer interactions: A review of progress in understanding polymer resist mask durability during plasma etching for nanoscale fabrication. *J. Vac. Sci. Technol.* **29**, 10801 (2011).

30. P. Amornsudthiwat, S. Damrongsakkul, Oxygen plasma etching of silk fibroin alters surface stiffness: A cell-substrate interaction study. *Plasma Processes Polym.* **11**, 763–776 (2014).

31. S. P. Timoshenko, J. M. Gere, *Theory of Elastic Stability* (McGraw-Hill, 1961).

32. S. Hirotsu, Electric-field-induced phase transition in polymer gels. *Jpn. J. Appl. Phys.* **24**, 396–388 (1985).

33. R. Kishi, M. Hasebe, M. Hara, Y. Osada, Mechanism and process of chemomechanical contraction of polyelectrolyte gels under electric field. *Polym. Adv. Technol.* **1**, 19–25 (1990).

34. J. Liu, J. Huang, T. Su, K. Bertoldi, D. R. Clarke, Structural transition from helices to hemihelices. *PLOS ONE* **9**, 93183–93189 (2014).

35. E. Jabbari, J. Tavakoli, A. S. Sarvestani, Swelling characteristics of acrylic acid polyelectrolyte hydrogel in a dc electric field. *Smart Mater. Struct.* **16**, 1614–1620 (2007).

36. W. Kuhn, B. Hargitay, A. Katchalsky, H. Eisenberg, Reversible dilation and contraction by changing the state of ionization of high-polymer acid networks. *Nature* **165**, 514–516 (1950).

37. R. Kishi, Y. Osada, Reversible volume change of microparticles in an electric field. *J. Chem. Soc. Faraday Trans.* **85**, 655–662 (1989).

38. T. Tanaka, Collapse of gels and the critical endpoint. *Phys. Rev. Lett.* **40**, 820–823 (1978).

39. S. Nemat-Nasser, J. Y. Li, Electromechanical response of ionic polymer-metal composites. *J. Appl. Phys.* **87**, 3321–3331 (2000).

40. P. J. Glazer, M. Van Erp, A. Embrechts, S. G. Lemay, E. Mendes, Role of pH gradients in the actuation of electro-responsive polyelectrolyte gels. *Soft Matter* **8**, 4421–4426 (2012).

41. T. Shiga, T. Kurauchi, Deformation of polyelectrolyte gels under the influence of electric field. *J. Appl. Polym. Sci.* **39**, 2305–2320 (1990).

42. T. Shiga, Y. Hirose, A. Okada, T. Kurauchi, Bending of ionic polymer gel caused by swelling under sinusoidally varying electric fields. *J. Appl. Polym. Sci.* **47**, 113–119 (1993).

43. Y. Osada, J.-P. Gong, Soft and wet materials: Polymer gels. *Adv. Mater.* **10**, 827–837 (1998).

44. Y. Osada, R. Kishi, M. Hasebe, Anomalous chemomechanical characteristics of electro-activated polyelectrolyte gels. *J. Polym. Sci. C Polym. Lett.* **25**, 481–485 (1987).

45. T. Tanaka, I. Nishio, S.-T. Sun, S. Ueno-Nishio, Collapse of gels in an electric field. *Science* **218**, 467–469 (1982).

46. P. K. Jha, J. W. Zwanikken, J. J. De Pablo, M. Olvera De La Cruz, Electrostatic control of nanoscale phase behavior of polyelectrolyte networks. *Curr. Opin. Solid State Mater. Sci.* **15**, 271–276 (2011).

47. M. Doi, M. Matsumoto, Y. Hirose, Deformation of ionic polymer gels by electric fields. *Macromolecules* **25**, 5504–5511 (1992).

48. P. J. Flory, *Principles of Polymer Chemistry* (Cornell Univ. Press, 1953).

49. D. Wang, M. D. Thouless, W. Lu, J. R. Barber, Generation of perversions in fibers with intrinsic curvature. *J. Mech. Phys. Solids* **139**, 103932 (2020).

50. J. Huang, J. Liu, B. Kroll, K. Bertoldi, D. R. Clarke, Spontaneous and deterministic three-dimensional curling of pre-strained elastomeric bi-strips. *Soft Matter* **8**, 6291–6300 (2012).

51. S. Liu, Z. Yao, K. Chiou, S. I. Stupp, M. O. De La Cruz, Emergent perversions in the buckling of heterogeneous elastic strips. *Proc. Natl. Acad. Sci. U.S.A.* **113**, 7100–7105 (2016).

52. J. Xu, H. Wan, Z. Fang, X. Peng, J. Sun, J. Liang, X. Wang, C. Lan, M.-B. Wu, N. Zheng, J. Liu, B. Wu, Continuous fabrication of Janus liquid crystal elastomer fibers with programmable actuation. *Nat. Commun.* **17**, 2378 (2026).

53. T. Shiga, Y. Hirose, A. Okada, T. Kurauchi, Electric field-associated deformation of polyelectrolyte gel near a phase transition point. *J. Appl. Polym. Sci.* **46**, 635–640 (1992).

54. F. C. Frank, The strength and stiffness of polymers. *Proc. R. Soc. Lond. A* **319**, 127–136 (1970).

55. M. C. Escobar, T. J. White, Fast and slow-twitch actuation via twisted liquid crystal elastomer fibers. *Adv. Mater.* **36**, e2401140 (2024).

56. B. Mehrafrooz, L. Yu, L. Pandey, Z. S. Siwy, M. Wanunu, A. Aksimentiev, Electro-osmotic flow generation via a sticky ion action. *ACS Nano* **18**, 17521–17533 (2024).

57. G. Li, Z. Liu, Q. Huang, Y. Gao, M. Regula, D. Wang, L. Q. Chen, D. Wang, Stable metal battery anodes enabled by polyethylenimine sponge hosts by way of electrokinetic effects. *Nat. Energy* **3**, 1076–1083 (2018).

58. B. S. Pivovar, W. H. Smyrl, E. L. Cussler, Electro-osmosis in Nafion 117, polystyrene sulfonic acid, and polybenzimidazole. *J. Electrochem. Soc.* **152**, A53–A60 (2004).

59. B. S. Pivovar, An overview of electro-osmosis in fuel cell polymer electrolytes. *Polymer* **47**, 4194–4202 (2006).

60. B. Mosadegh, P. Polygerinos, C. Keplinger, S. Wennstedt, R. F. Shepherd, U. Gupta, J. Shim, K. Bertoldi, C. J. Walsh, G. M. Whitesides, Pneumatic networks for soft robotics that actuate rapidly. *Adv. Funct. Mater.* **24**, 2163–2170 (2014).

61. E. Acome, S. K. Mitchell, T. G. Morrissey, M. B. Emmett, C. Benjamin, M. King, M. Radakovitz, C. Keplinger, Hydraulically amplified self-healing electrostatic actuators with muscle-like performance. *Science* **359**, 61–65 (2018).

62. Y. Wang, J. Sun, W. Liao, Z. Yang, Liquid crystal elastomer twist fibers toward rotating microengines. *Adv. Mater.* **34**, 2107840 (2022).

63. M. A. Berger, C. Prior, The writhe of open and closed curves. *J. Phys. A Math. Gen.* **39**, 8321–8348 (2006).

64. F. B. Fuller, The writhing number of a space curve. *Proc. Natl. Acad. Sci. U.S.A.* **68**, 815–819 (1971).

65. B. Audoly, Y. Pomeau, *Elasticity and Geometry: From Hair Curls to the Non-Linear Response of Shells* (Oxford Univ. Press, 2010).

66. A. C. Ugural, S. K. Fenster, *Advanced Strength and Applied Elasticity* (Elsevier, 1977).

67. C. A. Brackley, A. N. Morozov, D. Marenduzzo, Models for twistable elastic polymers in Brownian dynamics, and their implementation for LAMMPS. *J. Chem. Phys.* **140**, 135103–135112 (2014).

68. J. Lequieu, A. Córdoba, J. Moller, J. J. De Pablo, 1CPN: A coarse-grained multi-scale model of chromatin. *J. Chem. Phys.* **150**, 215102–215119 (2019).
